# First-in-Human Phase 1b/2a Trial Shows Safety with MEK 1/2 Inhibitor EDV2209 as A Novel Treatment for Spontaneous Subarachnoid Hemorrhage

**DOI:** 10.1007/s12975-026-01504-y

**Published:** 2026-10-30

**Authors:** Emelie P. Fungbrant, Jesper Peter Bömers, Helene Ravnholt Jensen, Kirsten Møller, Troels Halfeld Nielsen, Erik Kinnman, Diana N. Krause, Tiit Mathiesen, Lars Edvinsson

**Affiliations:** 1 Department of Clinical Medicine, University of Copenhagen, Copenhagen, Denmark; 2 Department of Neurosurgery, Copenhagen University Hospital- Rigshospitalet, Copenhagen, Denmark; 3 Department of Neuroanaesthesiology, Copenhagen University Hospital- Rigshospitalet, Copenhagen, Denmark; 4 Department of Neurosurgery, Odense University Hospital, Odense, Denmark; 5 Department of Clinical Sciences, Division of Experimental Vascular Research, Lund University, Lund, Sweden; 6 Department of Pharmaceutical Sciences, School of Pharmacy & Pharmaceutical Sciences, University of California, Irvine, CA United States of America; 7 Department of Clinical Neuroscience, Karolinska Institutet, Stockholm, Sweden

**Keywords:** Subarachnoid hemorrhage, delayed ischemic deficit, Drug trial, Safety

## Abstract

We report findings from a Phase 1/2a clinical trial (EUCT No. 2024-514590-22-00, STOP-DCI), an interventional, randomized, double-blind, placebo-controlled, multiple ascending-dose, first-in-human trial to determine safety and evaluate clinical outcome for the MEK 1/2 inhibitor EDV2209 in patients with severe spontaneous subarachnoid hemorrhage (SAH). In Phase 1b (*N* = 11), three cohorts received a single intracerebral ventricular (ICV) bolus (8.55 µg, 17–28.5 µg). In Phase 2a (*N* = 16), two cohorts were given three ICV injections of 28.5 µg EDV2209 or placebo over 18 h, beginning within 8 h of SAH onset.

The study found an equal distribution of adverse events between treatment and placebo with no treatment-associated safety concerns. Outcomes and mortality were as expected or better for patients with severe SAH in the patients treated with EDV2209. Pharmacokinetics were reproducibly stable in the CSF with no detectable levels of EDV2209 in plasma.

Treatment with EDV2209 was considered safe and was interpreted to provide potential benefit for improving patient outcome following severe SAH. These results provide a rationale to continue with a Phase 2b / 3 study powered to determine efficacy as well as safety.

**Supplementary Information:**

The online version contains supplementary material available at https://doi.org/10.1007/s12975-026-01504-y.

## Introduction

Subarachnoid hemorrhage (SAH) from a ruptured cerebral aneurysm is a rare form of hemorrhagic stroke with severe morbidity and high mortality that typically strikes people in mid-life (40–60 years old) [1]. Treatment comprises stabilization of vital parameters, optimization of oxygenation and fluid homeostasis, management of acute hydrocephalus, prevention of rebleeding by surgery on interventional procedures along with the calcium channel blocker nimodipine as standard treatment [2, 3]. Two-thirds of patients survive ictus and reach hospital. They are at risk for DCI, a feared, delayed phase of cerebral dysfunction which typically occurs 4–10 days after ictus [4]. DCI is associated with death or permanent severe neurological impairment in in 10–15% of all SAH patients while remaining patients may be affected by milder neurological morbidity, cognitive dysfunction, post-traumatic stress disorder and increased risk of late post-stroke dementia [4–7]. Despite optimal therapy, 3-month mortality in patients that reach hospital alive after severe SAH was 23–28% in recently reported cohorts [8, 9]. Moreover, morbidity is extensive with a favorable outcome only in 40% at three months [10].

DCI is the result of events initiated by early brain injury (EBI) after ictus. DCI is thus potentially preventable as the treatment window between ictus and manifest DCI is several days; treatment could begin early during neurocritical care prior to DCI occurrence and the onset of irreversible neurological injury. Despite some benefit of nimodipine, DCI has remained a major cause of morbidity. So far additional, preclinically promising pharmacological approaches to prevent DCI have failed clinical efficacy [11–14].

**Fig. 1:**
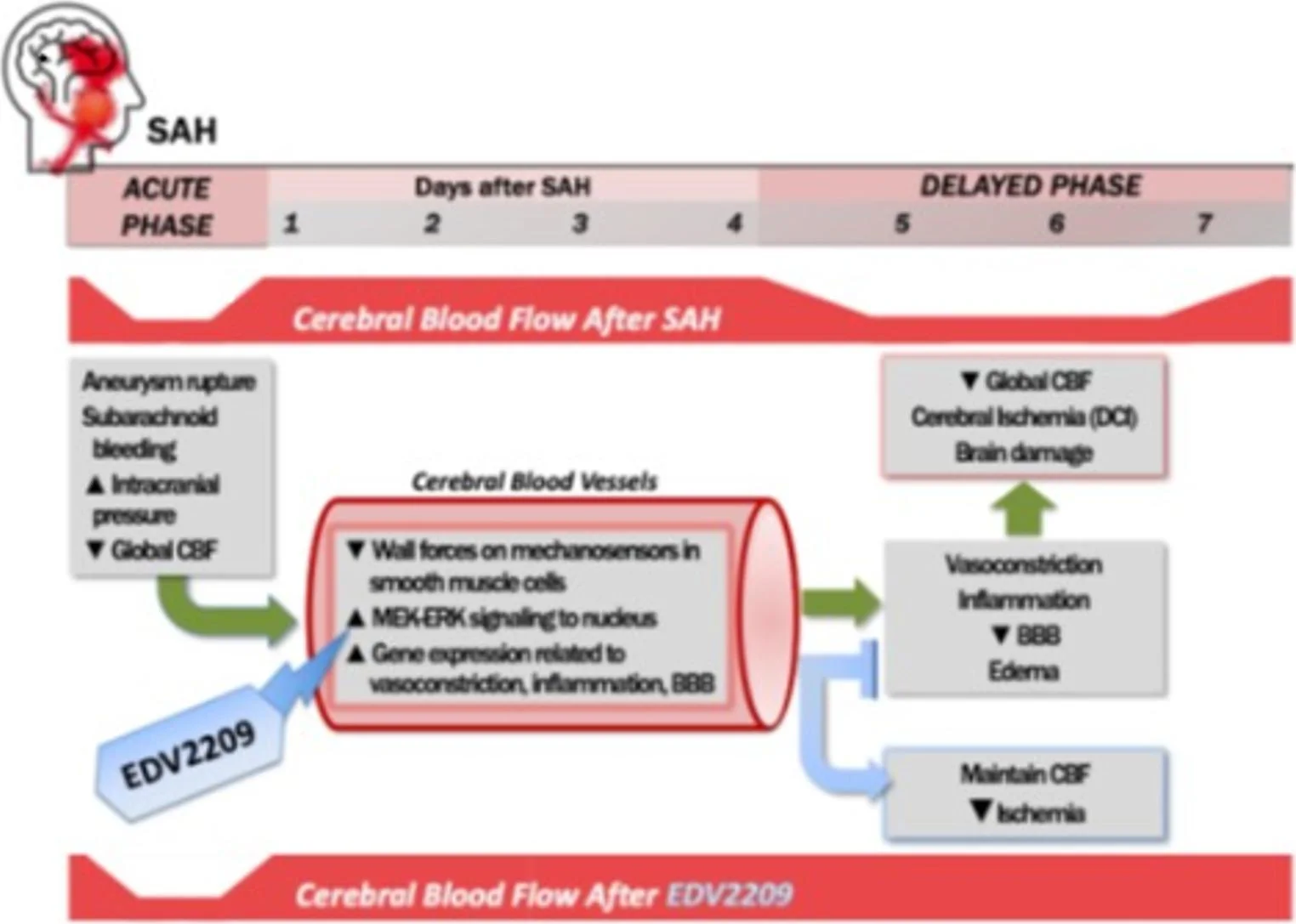
Proposed mechanism of action of EDV2209 in SAH [12]. In the first hours following aneurysm rupture (Acute Phase), there is subarachnoid bleeding, a sharp increase in intracranial pressure and significant decrease in global cerebral blood flow (CBF). Throughout the cerebral circulation, mechanical forces on the vascular wall are decreased, activating mechanosensors and intracellular signaling via the MEK-ERK pathway to the nucleus. During the days following, vascular genes related to vasoconstriction, inflammation and blood-brain barrier (BBB) dysfunction are switched on. This causes expression of pathophysiological changes during the Delayed Phase that result in a secondary drop in global blood flow, delayed global ischemia (DCI) and brain damage. ICV administration of EDV2209 given within 8 h of SAH onset blocks vascular MEK-ERK signaling and its consequences. This permits maintenance of proper CBF, prevention of DCI and improved outcome in the later phase

EDV2209 represents a novel approach for prevention of DCI and delayed brain damage following SAH (Fig. 1) [15]. It is based on the discovery that significant genomic changes occur throughout the brain vasculature in response to the suddenly increased intracranial pressure and subsequent decrease in cerebral blood flow following aneurysm rupture. Extensive preclinical studies have established that mechanosensors in the blood vessel wall activate intracellular MEK-ERK1/2 signaling within 6–8 h of ictus [15]. This pathway activates expression of multiple vascular genes involved in vasoconstriction, inflammation and blood-brain barrier integrity that contribute to the secondary drop in blood flow and cerebral ischemia that occurs several days later. Early administration of EDV2209 in preclinical SAH models prevents this vascular response and delayed cerebral ischemia; it improves neurological outcome significantly [15–17]. In addition, preclinical safety assessment did not show any toxicity concerns (Supplement 1)^18–27^. A similar dosing procedure has previously been used in another clinical trial [23] and direct instillation of drug into the brain has been used for many years to treat infections and brain tumors [24].

This article reports results from the first-in-human Phase 1b/2a trial with the primary objective to evaluate safety of the MEK 1/2 inhibitor, EDV2209, as a novel treatment for prevention of DCI in patients with severe aneurysmal subarachnoid hemorrhage. We included a preliminary assessment of clinical outcomes to secondarily inform on a possible rationale to continue drug development into phase 2b and 3.

## Methods

A Phase 1b/2a interventional, randomized, double-blind, placebo-controlled, multiple ascending-dose, first-in-human trial of EDV2209 was performed from January 2023 to January 2025 at the University Hospital of Copenhagen (Rigshospitalet), Copenhagen, Denmark and at Odense University Hospital, Odense, Denmark.

### Study Design, Blinding and Randomization

Single Dose Escalation of EDV2209 (Phase 1b).

- Cohort 1: 3 patients, open label, 8.55 µg (1 dose)
- Cohort 2: 4 patients, randomized 3:1 active: placebo, 17 µg (1 dose)
- Cohort 3: 4 patients, randomized 3:1 active: placebo, 28.5 µg (1 dose)

3 Doses over 18 h (Phase 2a)

- Cohort 4: 4 patients, randomized 3:1 active: placebo, 28.5 µg/dose. Sequential inclusion with a minimum interval of 1 week
- Cohort 5: 12 patients, randomized 3:1 active; placebo, 28.5 µg/dose. Continuous inclusion

All study- and clinical personnel except for pharmacy, PK-laboratory staff, lead pharmacokineticist and Fortrea safety delegate were blinded to whether vials contained active drug or placebo in cohorts 2–5.

Cohort 1 is open label with no placebo group. Patients in Cohorts 2, 3, 4 and 5 were randomized to either EDV2209 or placebo in a 3:1 ratio. A trial-independent statistician generated the Randomization Specification Document and randomization list for each cohort. Each trial site received a randomization list resulting in stratification by trial site. The randomization process was managed using sealed code envelopes. An unblinded pharmacist at the trial site) managed the allocation of randomized treatment.

Due to an unfortunate mix-up in the administration of 2 drug kits, 2 patients in Cohort 5 were excluded from the randomization design.

### Study Drug Dosage

Study drug dosage was based on a series of preclinical work in rat and dog as requested by authorities. The dose chosen was initially evaluated in rat [25]. From the effective dose in rat, we calculated CSF volume from rat to human. This dosage was tested in isolated human brain arteries and found effective [26, 27]. Efficacious doses in a rat model of SAH and interspecies scaling using volume and flow parameters of relevance for the route of administration, i.e., ventricle volume for initial concentration, CSF volume to calculate Cmax and CSF flow (= clearance from the site of action) to calculate exposure over time (AUC = dose/clearance, i.e., CSF flow). To obtain the same efficacious Cmax in human as in the rat, a 500-fold higher dose is needed since both the ventricle volume and the total CSF volume is 500-fold larger in human compared to rat. Hence the effective dose in human, based on Cmax, is estimated to 28.5 µg.

Inclusion criteria: Male or female patients with moderate or severe spontaneous (non-traumatic) SAH (World Federation of Neurosurgical Societies (WFNS) grade 1–5) diagnosed with computer tomography (CT), requiring an ICV drain, and able to undergo first ICV injection of trial medication within 8 h after symptom onset. (All patients had arterial SAH, although the explicit inclusion criterion was “spontaneous non-traumatic SAH.)

Exclusion criteria: Expected survival < 48 h, intraventricular or intracerebral blood in the absence of subarachnoid blood, any severe or unstable chronic or acute concomitant condition, evidence of previous major cerebral damage, pregnancy or breastfeeding.

All patients received standard neurointensive care for patients with SAH. Nimodipine was administered to prevent symptomatic vasospasm. Patients were under continuous monitoring of neurological condition and vital signs. Blood pressure and fluid balance were maintained within normal limits. All patients had acute hydrocephalus, managed by extra-ventricular drainage. Drainage started at 15 cmH¬20 and adapted to clinical condition and drainage volume; discontinuation was attempted after 5–10 days. Increased intracranial pressure was managed by diagnostic workup and drainage of CSF. Delayed ischemic deficit was defined by development of new neurological deficit or decrease by two points on the Glasgow coma scale that was not explained by hydrocephalus, electrolyte abnormalities, ventriculitis, rebleeding or surgical ischemia. Potential concomitant angiographic vasospasm was analyzed by CT-angiography or digital subtraction angiography. Delayed ischemia was managed by increased blood pressure towards mean arterial pressure of 110–130 mmHg and neuroradiological intervention if severe and refractory. The ruptured aneurysms were repaired by clipping or coiling. Eight patients also received tranexamic acid to prevent rebleeding (Table 1). Hyperthermia was managed with work-up for infection and symptomatic treatment.

### EDV2209 Administration (Fig. 2)

**Fig. 2:**
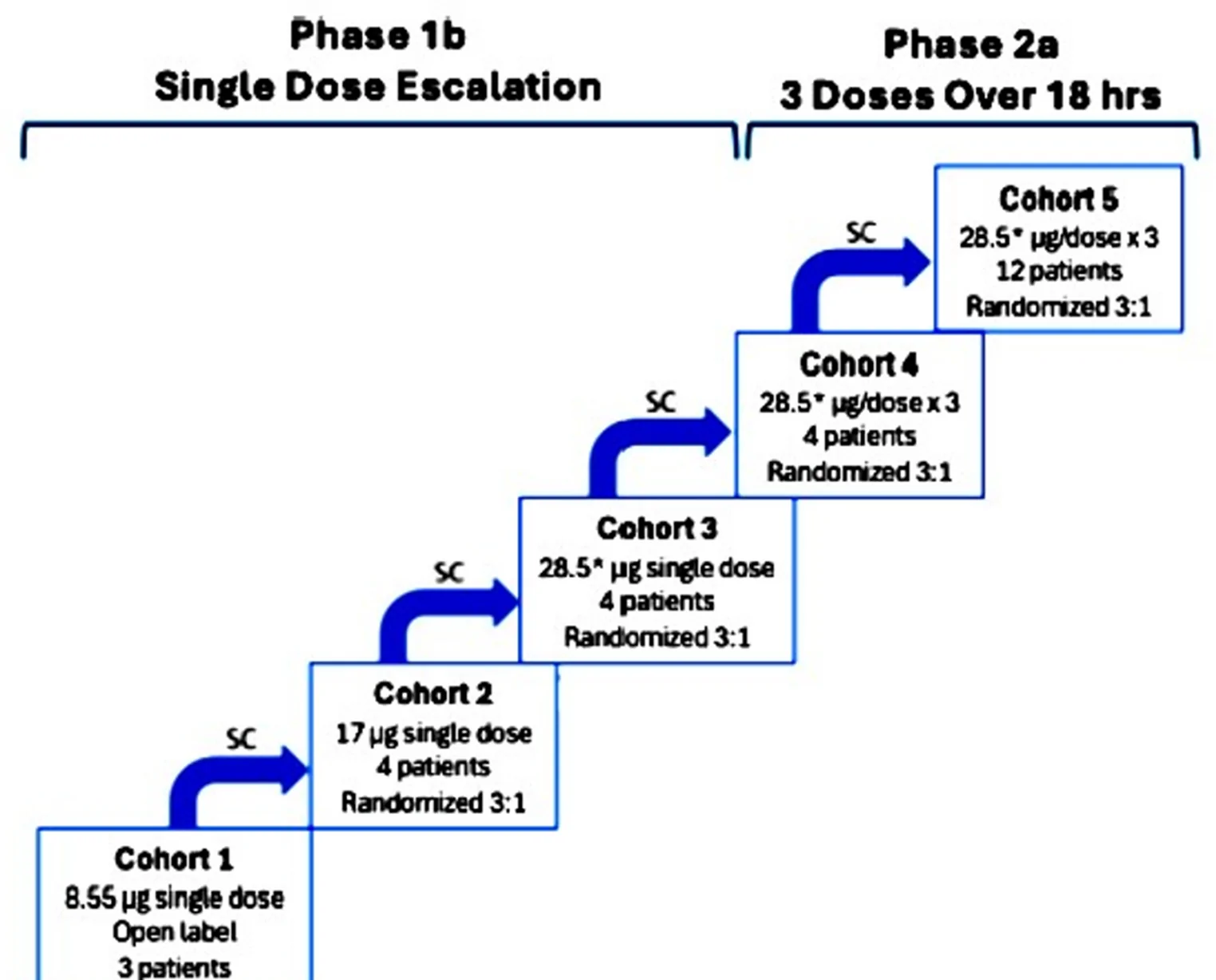
Study Design. Phase 1b was a Dose Escalation study to evaluate the safety of a single ICV bolus of EDV2209 in Cohort 1 (8.55 µg), Cohort 2 (17 µg drug or placebo) and Cohort 3 (28.5 µg drug or placebo). In Phase 2a, Cohorts 4 and 5 received three ICV injections of either 28.5 µg EDV2209 or placebo over a period of 18 h. Patients were assessed for any drug-related adverse events, CSF and plasma drug levels and functional outcome measures

EDV2209 (1,4-diamino-2,3-dicyano-1,4-bis (2-aminophenylthio) butadiene) was formulated and supplied in vials as a sterile solution for injection as an ICV bolus. The placebo consisted of 0.9% saline formulated in identical vials as EDV2209 and given in a similar manner.

Injections were undertaken under sterile conditions with continuous ICP monitoring. The EVD was kept and managed as a closed, sterile system. The injection volume is approximately 3 ml. Prior to injection, the same volume will be removed for PK assessment of EDV2209 in CSF. Therefore, intracranial volume does not increase.

Cohorts 1–3 (phase 1b) received a single injection within 8 h of the SAH ictus. Cohorts 4 and 5 (phase 2a) received 3 ICV bolus injections over 18 h in the early phase after SAH. The first dose was given within 8 h from the start of SAH symptoms. The second dose was given 6 h after the first dose, and the third dose given 12 h after the second dose.

### Primary Objective – Safety

Safety and tolerability of EDV2209 was assessed in patients with non-traumatic SAH who received at least one dose of drug. The patients were evaluated for possible treatment emergent adverse events (TEAEs), safety laboratory parameters for clinical chemistry, hematology, arterial blood gases, ECG, body weight, vital signs (blood pressure, heart rate, respiratory rate, body temperature), and neurological function. Phase 1b studied increasing ICV doses of the test drug in three cohorts (Fig. 2). After each administration, there was a pause of one week to review safety. After each cohort, results were evaluated by the safety committee and regulatory authorities prior to proceeding to the next cohort.

### Pharmacokinetic (PK) Measurements

Samples were collected via an external ventricular drain from the CSF and from plasma. CSF samples were immediately diluted by the addition of K_2_-EDTA human plasma at a ratio of 1:1 to form a matrix for the subsequent analysis. Sampling was done at the following time points: before each dose, 25 and 75 min after each dose and 12 h after the third dose.

Determination of EDV2209 in plasma and CSF was done using a specific bioanalytical method based on UPLC-MS/MS. The method was validated according to the EMA “Guideline on bioanalytical method validation” in the range of 1.00–1000 ng/mL for EDV2209 in CSF matrix, with a lower limit of quantification of 1.00 ng/ml. In plasma samples, the method was validated in the range of 0.500–500 ng/mL for EDV2209, with a lower limit of quantification of 0.500 ng/mL using a 100 µL plasma sample volume.

Measurements of EDV2209 in CSF included maximum (peak) concentration (C_max_) and nominal time for the occurrence of C_max_.

### Secondary Objective - Clinical Outcome

Clinical outcomes in all patients were measured by the Extended Glasgow Outcome Scale (GOS-E) [28, 29] and the Modified Rankin Scale [30] (mRS) at the time of discharge, 28 days and 84 days after ictus. With GOSE, higher scores indicate lower levels of disability. A GOSE score of 8 reflects no disability, scores in the 5–6 range indicate moderate disability whereas scores of 4 reflect severe disability; a favorable outcome was defined as GOSE > 4. In the mRS scale, lower scores indicate lower levels of disability (5 = severe, 4 = moderately severe, 3 = moderate, 2 = slight, 1 = no significant disability, 0 = no disability at all) [30]; a favorable outcome was defined as mRS < 3. The National Institute of Health Stroke Scale (NIHSS) [31] was recorded for patients alive on Day 14 or at discharge, whichever came first. The mean number of days at the Neuro-ICU from admittance until discharge was recorded for all patients in the study. The occurrence of DCI, new neurological deficits, and onset of vasospasm were considered safety issues and reported as adverse events or serious adverse events (Results and Supplement 4).

### Statistical Methods and Outcome Assessment

This is a first-in-human trial primarily designed to assess safety and tolerability. Vital signs, EKG, blood-tests and adverse events were followed and monitored by the principal investigator and a safety committee case-by-case. After study completion, cumulated adverse events were analyzed and compared to placebo.

As a Phase 1 safety trial, treatment was given sequentially in escalating cohorts to explore toxicity and side effects. Formal power-calculation is not feasible in the absence of previous data. Three patients received active treatment at each level and dose is escalated in the absence of toxicity; cohorts 2–5 included 1 placebo per 3 patients with active substance. Cohort 4 comprised four patients with sequential drug delivery of the highest dose one at a time while 9 patients in a final cohort of 12 patients received the active drug without additional observation of safety parameters before inclusion of a next patient. Disease progression and TEAEs were carefully monitored. The decision to include patients continuously was be made if safety and tolerability was judged to be favorable. The number of patients with active drug at the highest dosage was 12; a rule-of-thumb figure considered large enough to provide a reliable answer as accepted by the Danish Medicines Agency.

Patients with full intended treatment were compared to placebo to explore possible benefit and to support power calculation for an efficacy trial. Data from all patients in Cohorts 4 and 5 were combined with data from the 2 placebo patients in cohorts 2 and 3. Differences between the placebo (*n* = 5) and EDV2209 (*n* = 11) treatment groups in the outcome measures were evaluated with a dichotomized analysis of favorable and unfavorable outcomes for the Extended Glasgow Outcome scale (GOSE, 5–8 scores were considered favorable) and Modified Rankin Scale (mRS, scores of 0–2 were considered favorable). Descriptive statistics were used to describe patients while Fisher exact tests and Wilcoxon signed rank test were employed for comparative analyses; statistical significance was set at 0.05.

## Results

### Patients

A total of 27 eligible severe SAH patients were recruited and treated in the trial (Table 1) : 4 males (aged 47–68 years) and 23 females (aged 40–75 years). The cause of SAH was aneurysm rupture in all patients. The median WFNS score (World Federation of Neurological Surgeons grading scale for subarachnoid hemorrhage) was 4 at the time of admittance. 25 patients were recruited at the department of Neurosurgery, Copenhagen University Hospital, Denmark and 2 were recruited at the department of Neurosurgery, Odense, Denmark.

**Table 1:** Baseline data on patients as identified by cohort (1–5) and number within cohort (1–12)

Patient	Age (years)	Sex	Weight (kg)	WFNS Grade	Aneurysm localisation	Tranexamic acid
1–01	47	Female	65	II	Anterior communicating artery	No
1–02	57	Female	78	III	Basilar bifurcation	No
1–03	59	Female	61	V	Posterior communicating artery	Yes
2 − 01	41	Female	70	II	Anterior communicating artery	No
2–02	49	Female	60	III	Vertebral artery	No
2–03	55	Female	74	V	Anterior inferior cerebellar artery	Yes
2–04	61	Female	64	II	Posterior communicating artery	No
3 − 01	71	Female	75	IV	Anterior communicating artery	No
3 − 02	75	Female	70	IV	Middle cerebral artery	No
3–03	74	Female	70	IV	Superior Cerebellar artery	Yes
3–04	52	Female	67	V	Posterior communicating artery	No
4 − 01	49	Female	65	II	Posterior inferior cerebellar artery	Yes
4 − 02	57	Female	80	V	Posterior cerebral artery	Yes
4 − 03	58	Female	78	V	Vertebral artery	No
4–04	68	Male	59	II	Anterior communicating artery	No
5 − 01	52	Male	80	III	Middle cerebral artery	No
5 − 02	43	Female	75	I	Middle cerebral artery	No
5 − 03	47	Male	75	V	Posterior inferior cerebellar artery	Yes
5 − 04	53	Female	90	V	Middle cerebral artery	No
5–05	67	Female	78	IV	Basilar bifurcation	No
6–06	47	Female	75	IV	Posterior cerebral artery	No
5–07	51	Female	51	IV	Middle cerebral artery	Yes
5–08	55	Female	46	V	Anterior communicating artery	No
5–09	77	Female	86	IV	Anterior communicating artery	No
5–10	48	Female	65	II	Internal carotid arterty	Yes
5–11	66	Female	65	V	Posterior communicating artery	No
5–12	65	Female	78	V	Anterior communicating artery	No

### Condition at Admission of Placebo and Full Treatment Cohorts

Clinical condition at admission of cohorts 4 and 5 plus placebo patients from cohorts 2 and 3 was evaluated with the WFNS scale. 13 patients in cohorts 4 and 5 (mean age 57.0, median 55, range 43–77) received active drug: 9 had WFNS score 2–3 while 4 had a score of 4–5. Five patients received placebo (mean age 57.8, median 57, range 41–71): 4 had WFNS score 2–3 while 1 had a score of 4–5. The groups were similar (*p* = 1.0, Fisher test).

### Safety

#### Mortality

Four patients died within 84 days, one in cohort 2 (single dose of 17 µg active drug), one in cohort 3 (single dose of 28.5 µg active drug) and 2 in cohorts 4–5 (receiving three doses of 28.5 µg active drug). The cause of death in cohorts 2–3 was ischemic brain infarct at day 11 and day 14, respectively.

In cohorts 4–5, one patient died from rebleeding from an aneurysm that had not been fully secured at day 4 and one died from a parenchymal hemorrhage of the surgical site day 8 after ictus.

The deaths in cohorts 2–3 were considered the result of primary brain injury from the initial hemorrhage and the deaths in cohorts 4–5 were considered complications of surgical treatment.

No abnormalities with possible relation to systemic ischemia or hemorrhage were detected in blood tests. Mortality was not significantly different in placebo (*n* = 5) and actively treated patients (*n* = 23); *p* = 1.00, Odds Ratio = 0.3939; 95% CI: [0.0183, 8.4965]; the difference was also not significantly different when comparing placebo and active treatment in fully treated patients (cohorts 4 and 5): *p* = 1.00 and Odds Ratio = 0.657; 95% CI: [0.0251, 17.1811] The deaths were considered unrelated to the test drug by the safety committee.

#### Serious Adverse Events (Table 2)

Serious adverse events were reported in 3/5 placebo-patients (60%), 8/9 in the dose-finding cohorts 1–3 (89%) and 6/13 (44%) in full-treatment cohorts 4–5. Events included cerebral infarction or transient ischemia (*n* = 6), hydrocephalus, ventriculitis (*n* = 4), pneumonia or COPD exacerbation (*n* = 3), chronic subdural hematoma with aphasia and depressed level of consciousness (*n* = 1), pleural effusion (*n* = 1), shunt failure (*n* = 1), pancreatitis (*n* = 1), critical illness (*n* = 1), rebleeding from aneurysm (*n* = 1), intracranial parenchymal hemorrhage (*n* = 1).

Cerebral infarction led to death in two patients, both with associated pneumonia, aneurysmal rebleed led to death in one patient and intracranial parenchymal hemorrhage led to death in one patient. The patient with epilepsy recovered from SAH with residual epilepsy. One patient with a cerebral infarction recovered with sequelae as did the patient with critical illness. The remaining ten patients recovered from SAEs Table 3.

Neither the safety committee nor the principal investigator considered the events to be related to the trial medication; rather, they were considered expected for the cohort of SAH patients.

**Table 2:** Serious adverse events

Cohort Active/ Placebo	Event Description	Related to IMP	Outcome
1–01	Cerebral Infarction, Anteriorly On Left Side	Not related	Recovered with Sequelae
active	Pneumonia	Not related	Recovered
	Symptomatic Vasospasm Cerebral	Not related	Recovered
	Ventriculitis	Not related	Recovered
1–02	Transient Ischemic Attack	Not related	Recovered
active	Vertigo	Not related	Recovered
1–03	Confusion	Not related	Recovered
active	Hydrocephalus	Not related	Recovered
	Ventriculitis	Not related	Recovered
2 − 01 placebo	Ventriculitis	Not related	Recovered
2–02	Dysphagia	Not related	Recovered
active	Ventriculitis	Not related	Recovered
2–03	Cerebral Ischemic Infarction	Not related	Death
active	Pneumonia	Not related	Not recovered
3 − 01	Chronic Subdural Hematoma	Not related	Recovered
placebo	Depressed Level Of Consciousness	Not related	Recovered
	Expressive Aphasia	Not related	Recovered
	Sepsis	Not related	Recovered
3 − 02	COPD Exacerbation	Not related	Not recovered
active	Depressed Level Of Consciousness	Not related	Not recovered
	Malignant MCA Infarction	Not related	Death
3–03	Pain Thorax, Non-Cardiac	Not related	Recovered
active	Pleural Effusion Right And Left Side	Not related	Recovered
3–04	Cerebral edema	Not related	Recovered
active	Hydrocephalus Increased	Not related	Recovered
4 − 01	Epilepsy	Not related	Not recovered
active	Over drainage, Ventriculoperitoneal Shunt	Not related	Recovered
	Pancreatitis	Not related	Recovered
	Shunt Failure	Not related	Recovered
4 − 03 active	Critical Illness	Not related	Recovered with Sequelae
**5–05** placebo	Sepsis	Not related	Recovered
5–06 active	Infarction Intracranial Capsula Interna Right Side	Not related	Recovered
5–08 active	Rebleed From Aneurysm	Not related	Death
5–010	Delayed Cerebral Ischemia, Intermittent	Not related	Recovered
active	Pseudoaneurysm Left Leg	Not related	Recovered
5–11 active	Intracranial Parenchymal Haemorrhage	Not related	Death

**Table 3:** Summary of reported adverse events per treatment group; events may occur several times in one patient

Group	*N*	Not Serious			Serious		
		Not related	Possibly related	Total	Not related	Possibly related	Total
Active	22	581	6	587	31	0	31
Placebo	5	183	1	184	6	0	6
Total	27	764	7	771	37	0	37

#### Adverse Events (Table 4; Supplement)

Adverse events (AE) were equally distributed between treatment and placebo. The most common AE were obstipation, decrease in level of consciousness, prolonged QT-interval, headaches, electrolyte levels outside normal levels, decreased hemoglobin values, insomnia, nausea and vomiting. Ten patients with treatment were diagnosed with pulmonary infection, Transient elevation of liver enzymes was seen in 13 patients with active treatment and four with placebo.

Vital signs (Supplement 2) remained within normal limits without indications of systematically affected circulation, respiration or body temperature. No safety concerns arose that the safety committee judged to be related to the test drug EDV2209 in either the Phase 1b or Phase 2a parts of the trial.

**Table 4:** Reported AEs and SAEs

Category	Patients (27)		Events (808)	
	#	%	#	%
Reported AEs	27	100%	808	100%
Reported SAEs	17	63%	37	4.6%
Intensity = Mild	27	100%	370*	45.8%
Intensity = Moderate	25	93%	407**	50.4%
Intensity = Severe	13	48%	26	3.2%
Relation = Not Related	27	100%	801	99.1%
Relation = Possibly Related	3	11%	7	0.9%

*6 mild events were assessed as possibly related to IMP, for patient number

– 2501, increasing gamma-glutamyl transferase

– 2502, hyponatremia, increased alanine transaminase, aspartate transaminase, gamma glutamyl transferase, and increased lactate dehydrogenase

**1 moderate event was assessed as possibly related to IMP, for patient number

– 1302, hyponatremia

#### Safety of ICV Administration of Drug

No adverse events occurred that were related to the procedure of administration into the cerebroventricular catheter. Sustained ICP increase > 5 mmHg directly following injection was not seen. Early CSF infection within 5 days after ICV administration was not seen. During 2 weeks of treatment, ventriculitis was seen in 6 patients (22%; comparable to baseline expectations [32]).

### Pharmacokinetics (Tables 5 and 6; Fig. 3)

For patients in Cohorts 4 and 5, the concentration of EDV2209 in CSF was measured in samples taken after intraventricular drug administration (Fig. 3). After administration of each of 3 doses, maximum CSF concentrations were observed at the first sampling time post-administration (25 min) in 10 patients receiving EDV2209. The resultant geometric mean Cmax was 541 ng/mL after the first dose, 401 ng/mL after the second dose and 491 ng/mL after the last dose with an overall geometric mean of the highest Cmax observed across all three doses of 661 ng/ml. Thereafter, EDV2209 concentrations in CSF declined steadily, and by 720 min after the last dose, the concentrations were still quantifiable in all patients but relatively low (1.36 to 26.3 ng/mL). EDV2209 was not detected in patients receiving placebo Tables 4 and 5.

EDV2209 levels in plasma were analyzed at the same time points and were below the limit of quantification (0.500 ng/mL) for all subjects at all time-points.

**Table 5:** Geometric mean (Geometric%CV) PK parameters of EDV2209 following ICV Single Dose administration on Day 1 (first dose for last treatment)

Matrix	CSF	CSF	CSF	CSF	CSF
Parameter (Units)	8.55$\:\boldsymbol{\mu\:}$g SD Cohort 1 (*N*=3)	17$\:\boldsymbol{\mu\:}$g SD Cohort 2 With outlier (*N*=3)	17$\:\boldsymbol{\mu\:}$g SD Cohort 2 Without outlier (*N*=2)	28.5$\:\boldsymbol{\mu\:}$g SD Cohort 3 (*N*=3)	28.5$\:\boldsymbol{\mu\:}$g MD^e^ Cohort 4 and 5 (*N*=13)
AUC_0-last_^c^ (h*ng/mL)	970 (58.2%)	380 (779%)	1220 ^d^ (1100 – 1370)	1390 (29%)	586 (62.6%)
AUC_0-6_^c^ (h*ng/mL)	1720 ^a^	1190^d^ (1050 – 1350)	1190 ^d^ (1050 – 1350)	1190 (35.7%)	826 (61.0%)
AUC_0-12_^c^ (h*ng/mL)	1760 ^a^	1220^d^ (1100 – 1370)	1220^d^ (1100 – 1370)	1390 (29%)	NC
C0 (extrapolated) (ng/mL)	3860 (44.2%)	921 (1722%)	3590 ^d^ (2470 – 5230)	938 (50.6)	1140 (80.0%)
C_max_ (ng/mL)	699 (60.4%)	295 (555%)	861 ^d^ (780 – 950)	568 (44.2%)	541 (62.1%)
t_max_^b^ (h)	0.42 (0.42 – 0.42)	0.42 (0.42 – 0.42)	0.42 (0.42 – 0.42)	0.42 (0.42 – 0.42)	0.42 (0.42 – 0.42)
t_last_^b^ (h)	1.25 (1.25 – 12.00)	1.25 (1.25 – 12.00)	12.00 (12.00 – 12.00)	12.00 (12.00 – 12.00)	1.25 (1.25 – 1.25)
DAUC_0-last_^c^ (h*ng/mL/µg)	113 (58.2%)	22.3 (779%)	72.0 ^d^ (64.6 – 80.3)	48.9 (29%)	20.6 (62.6%)
DAUC_0-12_^c^ (h*ng/mL/µg)	206 ^a^	72.0 d (64.6 – 80.3)	72.0 ^d^ (64.6 – 80.3)	48.9 (29%)	NC
DC_max_ (ng/mL/µg)	81.8 (60.4%)	17.3 (555%)	50.6 ^d^ (45.9 – 55.9)	19.9 (44.2)	19.0 (62.1%)

^a^*N* = 1

^b^ Median (min-max) presented

^c^For some subjects, only 2 quantifiable postdose concentrations available, but AUCs reported to have the most possible data available for discussion

^d^Geomean (min-max) presented

^e^Parameter observed/calculated for the first administration profile

Bioanalysis was conducted for samples outside validated storage stability time for one subject in cohort 3 and for two subjects in cohort 4

NC = not calculated

DAUC_0-last_: AUC_0-last_ divided by the dose

DAUC_0-12_: AUC_0-12_ divided by the dose

DC_max_: C_max_ divided by the dose

**Table 6:** Dose proportionality of EDV2209 in CSF following single dose administration (Cohorts 1 to 3)

Dose	8.55 µg	17 µg# With outlier	17 µg Without outlier	28.5 µg#	28.5 µg
Dose increment	-	2.00	2.00	1.67	1.67
Increase in mean AUC_0-last_	-	0.39	1.26	3.66	1.14
DPF AUC_0-last_	-	0.20	0.63	2.19	0.68
Increase in mean C_max_	-	0.42	1.23	1.93	0.66
DPF C_max_	-	0.21	0.62	1.15	0.40

Dose proportionality based on preceding dose level

DPF = Dose Proportionality Factor – ratio of fold increase in geometric mean parameter divided by fold increase in dose

Dose proportionality for cohort 3 was reported for both with and without outlier of preceding dose level 17 µg

#Comparison to dose level 17 µg with outlier

**Fig. 3:**
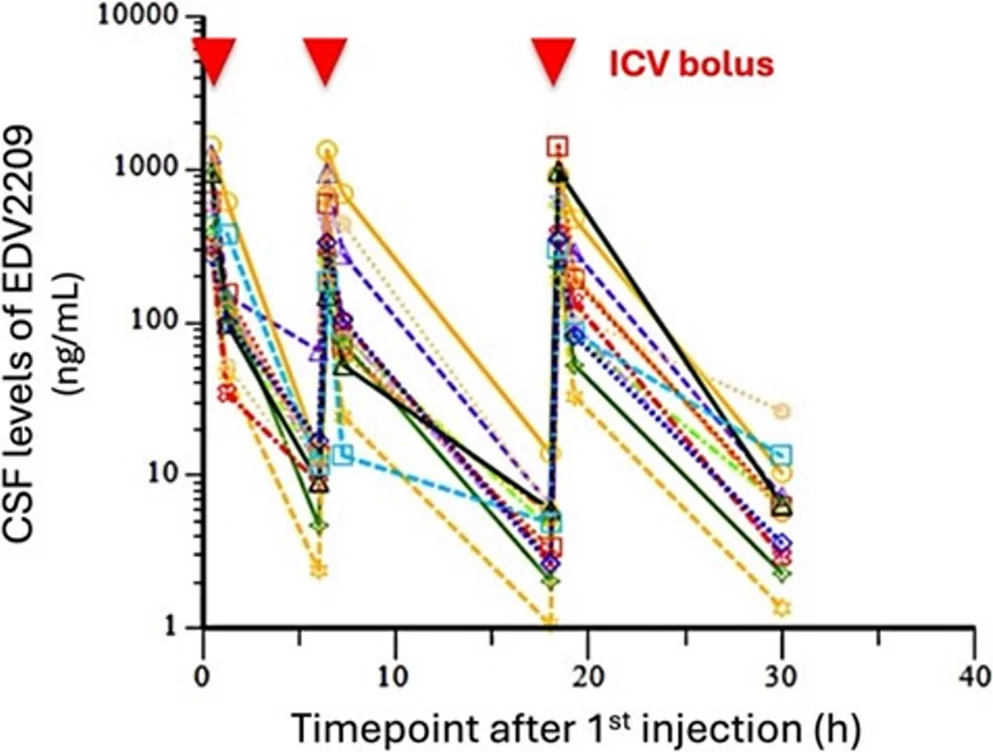
Individual CSF Concentrations of EDV2209 following ICV bolus injections of 28.5 µg EDV2209 at 3 time points. CSF samples were taken from SAH patients in Cohorts 4 and 5 over a 30-hour period

### Clinical Outcome Measures (Tables 7 and 8)

Assessment of clinical outcome was a secondary objective in Phase 2a. SAH patients from Cohorts 4 and 5 (given 3 doses of EDV2209, 28.5 µg per injection, or placebo over 18 h) were evaluated using the Extended Glasgow Outcome Scale (GOSE) at the time of discharge from neurosurgical care The two additional placebo-patients in cohorts 2 and 3 were included in analyses to improve power. Observed values are reported below. The differences between groups were not statistically significant.

**Table 7:** Dichotomized outcome according to the modified Rankin scale

Modified Rankin Scale.				
Group		Favorable = 0, 1 or 2		
		Discharge	Day 28	Day 84
Active				
	Favorable	1 (8%)	1 (8%)	9 (69%)
	Unfavorable	12 (92%)	12 (92%)	4 (31%)
	Total	13 (100%)	13 (100%)	13 (100%)
Placebo				
	Favorable	0 (0%)	1 (20%)	2 (40%)
	Unfavorable	5 (100%)	4 (80%)	3 (60%)
	Total	5 (100%)	5 (100%)	5 (100%)
P-value	Fisher test	1.00	0.49	0.33

**Table 8:** Dichotomized outcome according to the Extended Glasgow Outcome Scale

Extended Glasgow Outcome Scale.				
Group		Favorable = 5, 6, 7, 8		
		Discharge	Day 28	Day 84
Active				
	Favorable	6(46%)	6 (46%)	9 (69%)
	Unfavorable	7(54%)	7 (54%)	4 (31%)
	Total	13 (100%)	13 (100%)	13 (100%)
Placebo				
	Favorable	1 (20%)	2 (40%)	2 (40%)
	Unfavorable	4 (80%)	3 (60%)	3(60%)
	Total	5 (100%)	5 (100%)	5 (100%)
P-value	Fisher test	0.60	1.00	0.23

The GOSE values were median 5, range 1–8, mean 5.1 in the active treatment group (*n* = 13;; scores of 5–8 were considered favorable)) median 4, range 4–8, mean 5.0with placebo (*n* = 5). The mRS values in the active treatment group (*n* = 13) were median 1, range 0–6, mean 1.9 (scores of 0–2 were considered favorable)) and median 3, range 0–3, mean 2.0) with placebo (*n* = 5). Dichotomized values at discharge, days 28 and 84 are shown in Tables 7 and 8.

The 11 surviving SAH patients given EDV2209 were discharged from the hospital a median of 15 days after ictus (range 11–24; mean 16.3). Placebo patients (*n* = 5) were discharged after a median of 18 days (range 13–21, mean 16.8).

Symptomatic vasospasm / Delayed ischemic deficit was diagnosed in one patient with full active treatment (1/13) and in one placebo patient (1/5). An MRI or CT verified infarct was seen in 7 patients with active treatment (2/13 who received full active treatment and 5/9 during dose escalation). One/5 placebo patients had an imaging-verified infarct.

## Discussion

Advancements in treatment of SAH have primarily focused on early management of the patients with better diagnosis, prevention of rebleeding by early repair of the ruptured aneurysm, hydrocephalus management, advanced intensive care support and administration of nimodipine [1, 14]. Yet, there is a need for better pharmacological alleviation of DCI. Our study indicates a favorable safety profile with intraventricular EDV2209, a Raf-MEK inhibitor with promising preclinical effects on acute SAH.

### Safety and Feasibility

The trial targeted patients with severe SAH that required treatment with ventricular drainage. It was possible to place a ventricular drainage and include patients to receive the potentially neuroprotective drug into the cerebral ventricular system to bypass the blood-brain barrier (BBB) within 8 h after ictus. Adverse events and severe adverse events were balanced between active treatment and placebo and specific treatment related adverse events were not detected.

The favorable safety findings agreed with expectations. Raf-MEK1/2 inhibition drugs have been available for anti-cancer therapy. One such drug is trametinib, a MEK1/2 inhibitor indicated for the treatment of metastatic melanoma. Safety data following a Phase III trial in melanoma reported rash (57%), diarrhea (43%), peripheral oedema (26%), fatigue (26%) and acneiform dermatitis (19%), nausea (18%), alopecia (17%), hypertension (15%), constipation (14%) and vomiting (13%) as the most common side-effects of longer-term treatment [33]. QTc prolongation was not reported using trametinib as a single agent [33, 34]. The patients received continuous treatment until disease progression, death or withdrawal from the study. Safety data of short-term treatment are not available, although exposure limited to one day would cause fewer or no side effects from sustained catabolic interference with MEK-signaling; moreover, systemic presence of the drug was not detectable in our patients.

Intra-cerebroventricular drug administration is feasible in an intensive care setting, especially for patients who have an external ventricular drainage for clinical reasons. We replaced 3 ml of CSF with 3 ml of drug and thereby avoided expanding intracranial volume that might have affected intracranial pressure. Similar dosing procedures have been used previously [23, 24]. Neither previous authors nor we experienced safety problems with drug delivery.

### Mortality

Three month-mortality was 15.3% in patients with full 3-dose treatment and 22.2% in the single dose escalation cohorts. Mortality was judged by the safety committee and PI to be caused by severity of initial SAH (cerebral ischemia; *n* = 2) or complications to treatment (rebleeding of aneurysm and parenchymal hemorrhage; *n* = 2). Importantly, mortality was comparable to or below what was expected with causes consistent with those reported previously from our department [35]. Previous studies reported 24–26% mortality in SAH patients treated with EVD and 50% mortality in unconscious SAH patients who reach hospital after severe SAH [36, 38]. We recently found 23% mortality in SAH patients treated with ventricular drainage at our department [39].

The adverse and severe adverse events that were encountered in our SAH patients were similar to those typically seen in patients with severe SAH including disorders of consciousness, neurological symptoms, electrolyte aberrations, gastrointestinal symptoms, headaches and infections. They were distributed equally between treatment groups and placebo; typically, the adverse events resolved during follow-up. The safety committee, sponsor or PI could not identify any adverse events associated with treatment. It is possible that treatment with tranexamic acid, which was previously recommended in Danish Guidelines, could have added to risk of hemorrhagic complications. The small numbers of patients did not allow targeted analyses, and future management will not be confounded by tranexamic acid, as treatment recommendations have been updated. Typical class-related TEAE for MEK/ERK inhibitors such as rash, alopecia, fatigue, diarrhea, peripheral edema, and nausea were not associated with active treatment. Moreover, systemic exposure to the experimental drug was not measurable, further strengthening the assessment of systemic safety. Neither transient hepatic enzyme elevations nor pulmonary infections were statistically associated with treatment. These AEs were not severe but need to be further monitored in continued studies.

### Clinical Outcome

The study was not powered for efficacy and assessment of a possible drug effect was considered exploratory and a useful guide for design of a phase 2b / 3 trial. Nonetheless, groups were well balanced in terms of SAH severity and ages, and simple comparisons were compatible with, or even suggested, better outcomes in terms of GOSE and mRS scores in active treatment compared with placebo. The differences were not statistically significant but provided a favorable signal to extend efficacy assessment beyond this pilot phase. Moreover, a comparison with historical controls suggested benefit in terms of a favorable GOSE score for 70% of patients receiving active treatment compared to 33% in literature [10]. The rudimentary assessment of outcome was solely intended to support decision-making on the rationality to continue drug development and could neither prove nor exclude clinical efficacy. An outcome study will need to be powered for a clinical primary outcome and include similar secondary outcomes as previous trials on neuroprotection after SAH: occurrence of delayed ischemic deficit and late MRI-verified infarcts [2, 12, 40, 41].

### Rationale for Continuation

There is a clinical need for pharmacological therapies to ameliorate morbidity and mortality as well as prevent loss of cognitive and motor functions following SAH. Hemorrhagic stroke due to aneurysm rupture is a devastating illness with a high rate of mortality and long-term disability [1]. Recent statistics show a global age adjusted incidence of 8.3 (95% UI, 7.3–9.5) and a DALY rate 125.2 (95% UI, 110.5-142.6) per 100 000 people. The latter reflects the tremendous number of years lost to premature death or disability due to SAH. Subarachnoid hemorrhage is much less common than intracerebral hemorrhages and ischemic stroke, but occurs disproportionally in younger populations,40–70 years of age, causing a relatively larger loss of quality adjusted life years. The absolute case numbers increased from 1990 to 2021 and SAH ranked as the 36th most common cause of death in the world [35].

It is generally accepted that the secondary brain injury that develops during the first 72 h after SAH is the most influential clinical determinant of outcome when patients have survived ictus [11, 13, 42]. Current research is focused on understanding the pathophysiology underlying the delayed phase of cerebral ischemia (DCI) and early brain injury following SAH. Multiple processes are involved including decreased cerebral blood flow, global brain ischemia, inflammation, mitochondrial dysfunction, cortical spreading depolarization, microcirculatory dysfunction, blood-brain-barrier breakdown, cerebral edema, oxidative cascades, and neuronal death [11, 13, 42]. Many potential targets have been identified, however, none of the recent trials have resulted in new treatments for SAH^11^. For over 30 years, oral nimodipine has been the only drug specifically recommended for preventing late morbidity of SAH [11, 13]. Nimodipine is a vasodilator originally developed as a means to prevent vasospasm after SAH [2]. Clinical studies of nimodipine show at best modest efficacy in improving SAH outcome, and DCI remains an important cause of morbidity and disabilities. Better or additional treatments are needed.

It is our view that an early biological “switch”, such as the ras-raf-MEK1/2-ERK1/2 pathway that triggers multiple changes in the cerebral vasculature that evolve over time, is an important target [18–22]. A key outcome in preclinical studies was preservation of global cerebral blood flow (CBF) in the delayed phase to prevent brain ischemia and its deleterious effects [15]. Numerous animal studies of experimental SAH have shown that inhibition of the MAP kinase pathway within 8 h of stroke onset maintains CBF at later time points and significantly improves neurological function [15–17, 43, 44, 46]. The overall aim of the current Phase 1/2a trial of EDV2209 was to investigate clinical feasibility and safety of a novel treatment strategy developed over 25 years of preclinical studies (Fig. 1) [15]. The study showed safety of intra-ventricular EVD2209 treatment and a favorable pharmacokinetic profile. A phase 1/2a trial cannot evaluate efficacy. Yet, a decision on whether a continuation of drug development is justified ethically would require an analysis of expected benefit, preferably beyond the expectations from preclinical studies. We made an exploratory comparison to historical literature data (figure in supplement; Trizio et al. ^10^). Trizio et al. analyzed a comparable cohort of SAH patients and found an unfavorable outcome at 3 months after ictus in 66%, while only a minority, 30%, of our patients had a similarly unfavorable outcome. Taken together, we conclude that observations were sufficiently promising to continue development towards a trial designed to evaluate efficacy.

## Conclusion

Intraventricular treatment with EDV2209 was found safe in a first-in-humanPhase1/2a trial. Comparisons between active treatment and placebo groups or historical controls were not powered for statistical significance. Yet, exploration of outcomes was considered to support continued drug development towards efficacy trials. These encouraging findings may lead the way to developing effective SAH therapies that prevent the serious consequences of DCI by targeting MEK-signaling.

## Supplementary Information

Below is the link to the electronic supplementary material.

Supplementary Material 1

## Data Availability

All data supporting the findings of this study are available within the paper and its Supplementary Information.

## References

[1] Claassen J, Park S. Spontaneous subarachnoid haemorrhage. Lancet. 2022;400:846–62. 10.1016/S0140-6736(22)00938-2. PMC9987649 35985353

[2] Allen GS, et al. Cerebral arterial spasm–a controlled trial of nimodipine in patients with subarachnoid hemorrhage. N Engl J Med. 1983;308:619–24. 10.1056/NEJM198303173081103. 6338383

[3] Macdonald RL, Schweizer TA. Spontaneous subarachnoid haemorrhage. Lancet. 2017;389:655–66. 10.1016/S0140-6736(16)30668-7. 27637674

[4] Macdonald RL. Delayed neurological deterioration after subarachnoid haemorrhage. Nat Rev Neurol. 2014;10:44–58. 10.1038/nrneurol.2013.246. 24323051

[5] Dreier JP, et al. Spreading depolarizations in ischaemia after subarachnoid haemorrhage, a diagnostic phase III study. Brain. 2022;145:1264–84. 10.1093/brain/awab457. 35411920

[6] Dreier JP, et al. All Three Supersystems-Nervous, Vascular, and Immune-Contribute to the Cortical Infarcts After Subarachnoid Hemorrhage. Transl Stroke Res. 2025;16:96–118. 10.1007/s12975-024-01242-z. PMC11772491 38689162

[7] Corraini P, et al. Long-Term Risk of Dementia Among Survivors of Ischemic or Hemorrhagic Stroke. Stroke. 2017;48:180–6. 10.1161/STROKEAHA.116.015242. PMC5796652 27899749

[8] Stauning AT, Eriksson F, Benndorf G, Holst AV, Hauerberg J, Stavngaard T, Poulsgaard L, Rochat P, Eskesen V, Birkeland P, Mathiesen T, Munch TN. Mortality among patients treated for aneurysmal subarachnoid hemorrhage in Eastern Denmark 2017–2019. Acta Neurochir (Wien). 2022;164(9):2419–30. 10.1007/s00701-022-05303-w. PMC9303843 35864221

[9] An X, Cai X, Wang R, Wang Y, Liu Z, Dong W, Sun Y, Zhao Y, Yang X, Chai Y, Guo F. Comparison of microsurgical and endovascular treatment outcomes in poor-grade aneurysmal subarachnoid hemorrhage: a multicenter cohort study. Neurosurg Rev. 2026;49(1):210. 10.1007/s10143-026-04167-3. 41656448

[10] de Trizio I, Ferrario A, Bogli SY, Casagrande F, Garcia Alzamora M, Sebok M, et al. Longitudinal trajectories of functional outcome following aneurysmal subarachnoid hemorrhage: A retrospective study. Crit Care. 2026;30(1):59. 10.1186/s13054-025-05808-7. PMC12874848 41507948

[11] Yang BSK, et al. Update on Strategies to Reduce Early Brain Injury after Subarachnoid Hemorrhage. Curr Neurol Neurosci Rep. 2024;25:14. 10.1007/s11910-024-01396-1. PMC13081737 39722093

[12] Caylor MM, Macdonald RL. Pharmacological Prevention of Delayed Cerebral Ischemia in Aneurysmal Subarachnoid Hemorrhage. Neurocrit Care. 2023. 10.1007/s12028-023-01847-6. 37740138

[13] Osgood ML. Aneurysmal Subarachnoid Hemorrhage: Review of the Pathophysiology and Management Strategies. Curr Neurol Neurosci Rep. 2021;21:50. 10.1007/s11910-021-01136-9. 34308493

[14] Neifert SN, et al. Aneurysmal Subarachnoid Hemorrhage: the Last Decade. Transl Stroke Res. 2021;12:428–46. 10.1007/s12975-020-00867-0. 33078345

[15] Edvinsson L, Krause DN, Switching Off Vascular MAPK. Signaling: A Novel Strategy to Prevent Delayed Cerebral Ischemia Following Subarachnoid Hemorrhage. Transl Stroke Res. 2024. 10.1007/s12975-024-01234-z. PMC12045832 38334872

[16] Bomers JP, Grell AS, Edvinsson L, Johansson SE, Haanes KA. The MEK inhibitor trametinib improves outcomes following subarachnoid haemorrhage in female rats. Pharmaceuticals (Basel). 2022;15. 10.3390/ph15121446. PMC9785726 36558896

[17] Larsen CC, Povlsen GK, Rasmussen MN, Edvinsson L. Improvement in neurological outcome and abolition of cerebrovascular endothelin B and 5-hydroxytryptamine 1B receptor upregulation through mitogen-activated protein kinase kinase 1/2 inhibition after subarachnoid hemorrhage in rats. *J Neurosurg* 114, 1143–1153 (2011). 10.3171/2010.6.JNS1018 endovascular coiling. Stroke. 2012;43(6):1463-9. 10.1161/STROKEAHA.111.648980 20597604

[18] Edvinsson L, Povlsen GK. Vascular plasticity in cerebrovascular disorders. J Cereb Metab Blood Flow. 2011;31:1554–71. 10.1038/jcbfm.2011.70 PMC3137480 21559027

[19] Povlsen GK, Edvinsson L. MEK1/2 inhibitor u0126 but not endothelin receptor antagonist clazosentan reduces upregulation of cerebrovascular contractile receptors and delayed cerebral ischemia, and improves outcome after subarachnoid hemorrhage in rats. J Cereb Blood Flow Metab. 2015;35(2):329–37. 10.1038/jcbfm.2014. PMC4426751 25407271

[20] Edvinsson L, Larsen SS, Maddahi A, Nielsen J. Plasticity of cerebrovascular smooth muscle cells after subarachnoid hemorrhage. Transl Stroke Res. 2014;5(3):365–76. 10.1007/s12975-014-0331-4. 24449486

[21] Povlsen GK, Johansson SE, Larsen CC, Samraj AK, Edvinsson L. Early events triggering delayed vasoconstrictor receptor upregulation and cerebral ischemia after subarachnoid hemorrhage. BMC Neurosci. 2013;14:34. 10.1186/1471-2202-14-34. PMC3618233 23496889

[22] Mostajeram M, Edvinsson L, Warfvinge K, Singh R, Ansar S. Inhibition of mitogen-activated protein kinase 1/2 in the acute phase of stroke improves long-term neurological outcome and promotes recovery processes in rats. Acta Physiol (Oxford). 2017;219(4):814–24. 10.1111/apha.12632 26595054

[23] Cook AM, Mieure KD, Owen RD, Pesaturo AB, Hatton J. Intracerebroventricular administration of drugs. Pharmacotherapy. 2009;29(7):832–45. 10.1592/phco.29.7.832 19558257

[24] Thomas JE, Rosenwasser RH, Armonda RA, Harrop J, Mitchell W, Galaria I. Safety of intrathecal sodium nitroprusside for the treatment and prevention of refractory cerebral vasospasm and ischemia in humans. Stroke. 1999;30(7):1409–16. 10.1161/01.str.30.7.1409 10390315

[25] Larsen CC, Povlsen GK, Rasmussen MN, Edvinsson L. Improvement in neurological outcome and abolition of cerebrovascular endothelin b and 5-hydroxytryptamine 1b receptor upregulation through mitogen-activated protein kinase kinase 1/2 inhibition after subarachnoid hemorrhage in rats. J Neurosurg. 2011;114(4):1143–53. 10.3171/2010.6.JNS1018. 20597604

[26] Ansar S, Eftekhari S, Waldsee R, Nilsson E, Nilsson O, Saveland H, et al. Mapk signaling pathway regulates cerebrovascular receptor expression in human cerebral arteries. BMC Neurosci. 2013;14:12. 10.1186/1471-2202-14-12. PMC3663811 23343134

[27] Ahnstedt H, Saveland H, Nilsson O, Edvinsson L. Human cerebrovascular contractile receptors are upregulated via a b-raf/mek/erk-sensitive signaling pathway. BMC Neurosci. 2011;12:5. 10.1186/1471-2202-12-5. PMC3023719 21223556

[28] Mehta R, Chinthapalli K. Glasgow coma scale explained. BMJ. 2019;365:l1296. 10.1136/bmj.l1296. 31048343

[29] Teasdale G, Jennett B. Assessment and prognosis of coma after head injury. Acta Neurochir (Wien). 1976;34(1–4):45–55. 10.1007/BF01405862 961490

[30] Broderick JP, Adeoye O, Elm J. Evolution of the Modified Rankin Scale and Its Use in Future Stroke Trials. Stroke. 2017;48:2007–12. 10.1161/STROKEAHA.117.017866. PMC5552200 28626052

[31] Adams HP Jr., et al. Baseline NIH Stroke Scale score strongly predicts outcome after stroke: A report of the Trial of Org 10172 in Acute Stroke Treatment (TOAST). Neurology. 1999;53:126–31. 10.1212/wnl.53.1.126. 10408548

[32] Dorresteijn KRIS, Jellema K, van de Beek D, Brouwer MC. Factors and measures predicting external CSF drain-associated ventriculitis: A review and meta-analysis. Neurology. 2019;93(22):964–72. 10.1212/WNL.0000000000008552. 31659095

[33] Flaherty K, METRIC Study Group. Improved survival with MEK inhibition the BRAF-mutated melanoma. N Engl J Med. 2012a;367:107–14. 10.1056/NEJMoa1203421 22663011

[34] Flaherty K, Infante J, Daud A, Gonzalez R, Kefford R, Sosman J, Engl, et al. J Med. 2012b;367:1694–703. 10.1056/NEJMoa1210093 PMC3549295 23020132

[35] GBD 2021 Global Subarachnoid Hemorrhage Risk Factors Collaborators; Rautalin I, Volovici V, Stark BA, Johnson CO, Kaprio J, Korja M, Krishnamurthi RV, Nair BS, Ranta A, Rinkel GJE, Vergouwen MDI, Abate YH, Abbastabar H, Abd-Allah F, Abdelkader A, Abdi P, Abdollahi A, Abdullahi A, Abiodun OO, Aboagye RG, Abouzid M, Abtahi D, Abu Rumeileh S, Abualhasan A, Abualruz H, Abukhadijah HJ, Abu-Zaid A, Adamu LH, Addo IY, Adedoyin RA, Adegboye OA, Adra S, Adzigbli LA, Agyemang-Duah W, Ahinkorah BO, Ahmad A, Ahmad D, Ahmadzade AM, Ahmed A, Ahmed H, Ahmed SA, Aji B, Akkaif MA, Al-Ajlouni Y, Al-Aly Z, Albashtawy M, Ali MU, Alif SM, Alimohamadi Y, Aljunid SM, Alomari MA, Alrawashdeh A, Alsabri MA, Salman RA, Altaf A, Al-Tammemi AB, Alvis-Guzman N, Alwafi H, Al-Wardat M, Al-Worafi YM, Aly H, Alyahya MSI, Alzoubi KH, Amani R, Amin TT, Amindarolzarbi A, Amusa GA,Anderlini D, Angappan D, Anil A, Anuoluwa BS, Anwar S, Anyasodor AE, Apostol GLC,Arabloo J, Areda D, Ärnlöv J, Artamonov AA, Artanti KD, Arumugam A, Aryan Z, Asghari-Jafarabadi M, Ashemo MY, Ashraf T, Athar M, Athari SS, Aujayeb A, Awotidebe AW, Azadnajafabad S, Aziz S, Azzam AY, Babu GR, Bagheri N, Bahrami Taghanaki P, Bahramian S, Bai R,Baig AA, Bako AT, Baltatu OC, Bam K, Banach M, Bandyopadhyay S, Banik B, Bardhan M,Barker-Collo SL, Bärnighausen TW, Barqawi HJ, Barua L, Bastan MM, Basu S, Bell SL,Bensenor IM, Berhie AY, Beyene KA, Bhagavathula AS, Bhaskar S, Bhat AN, Bhat V, Bhatti GK, Bhatti JS, Bijani A, Bikbov B, Birhan MM, Birhanu MM, Bitra VR, Boloor A, Borhany H, Breitner S, Brenner H, Bugiardini R, Bulamu NB, Butt ZA, Cabral LS, Caetano Dos Santos FL, Calina D, Cámera LA, Campos LA, Campos-Nonato I, Capodici A, Carvalho F,Castañeda-Orjuela CA, Catapano AL, Cegolon L, Chadwick J, Chakraborty C, Chakraborty PA, Chakraborty S, Chandika RM, Chanie GS, Chattu VK, Chaudhary AA, Chi G, Chichagi F, Ching PR, Chopra H, Choudhari SG, Chowdhury EK, Chu DT, Chung SC, Columbus A, Criqui MH, da Silva AG, Dabbagh Ohadi MA, Dadras O, Dai X, Dalal K, Dalli LL, D’Amico E,Dashti M, Davletov K, De la Cruz-Góngora V, Debopadhaya S, Delgado-Enciso I, Derviševic E, Devanbu VGC, Dewan SMR, Dhane AS, Dibas M, Do TC, Do THP, Dohare S, Doheim MF,Dokova KG, Dongarwar D, D’Oria M, Doshi OP, Doshi RP, Dowou RK, Dsouza HL, Dutta S,Dziedzic AM, E’mar AR, Edvardsson D, Efendi D, Efendi F, El Nahas N, Elgendy IY, Elhadi M, Eltaha C, Eltahir ME, Emeto TI, Fabin N, Fagbamigbe AF, Fahim A, Fakhradiyev IR,Fares J, Faris PS, Fauk NK, Fazylov T, Fekadu G, Ferreira N, Fetensa G, Fischer F,Foschi M, Fridayani NKY, Gaipov A, Gajjar AA, Gandhi AP, Ganesan B, Garg RK, Gebregergis MW, Gebrehiwot M, Gebremeskel TG, Getie M, Ghadimi DJ, Ghadirian F, Ghahramani S,Ghasemzadeh A, Ghazy RM, Gholamalizadeh M, Ghozy S, Gil AU, Gilani JA, Gnedovskaya EV, Goleij P, Goulart AC, Goulart BNG, Guan SY, Gupta S, Habibzadeh F, Hadei M, Hadi NR, Hamidi S, Hanifi N, Hankey GJ, Harlianto NI, Haro JM, Hasan F, Hasani H, Hasnain MS, Hassan Zadeh Tabatabaei MS, Haubold J, Havmoeller RJ, Hay SI, Hbid Y, Heidari G, Heidari M, Hemmati M, Hiraike Y, Hoan NQ, Holla R, Hosseinzadeh M, Hostiuc S, Huang J, Huynh HH, Hwang BF, Ibitoye SE, Ikeda N, Ikiroma A, Ilaghi M, Ilesanmi OS, Ilic IM, Ilic MD, Islam MR, Ismail NE, Iso H, Isola G, Iwagami M, Jacob L, Jafarzadeh A,Jain A, Jairoun AA, Jakovljevic M, Jatau AI, Jawaid T, Jayapal SK, Jonas JB, Joseph N, Jürisson M, Kadashetti V, Kalani R, Kamal VK, Kamireddy A, Kanchan T, Kandel H,Karami J, Karaye IM, Karimi Y, Karimi Behnagh A, Kashoo FZ, Kayode GA, Kazemi F, Kesse-Guyot E, Khader YS, Khaing IK, Khan F, Khan MJ, Khatatbeh H, Khatatbeh MM, Khayat Kashani HR, Kheirallah KA, Khidri FF, Khormali M, Khosla AA, Kim K, Kim YJ, Kisa A, Kisa S,Kivimäki M, Kolahi AA, Kompani F, Korzh O, Kostev K, Kothari N, Krishan K, Krishna V, Krishnamoorthy V, Kuddus M, Kulimbet M, Kunutsor SK, Kurniasari MD, Kusuma D, Kytö V, La Vecchia C, Lahariya C, Lai DTC, Lai H, Laksono T, Lallukka T, Latief K, Latifinaibin K, Le NHH, Le TTT, Lee M, Lee SW, Lee WC, Lee YH, Lenzi J, Leonardi M, Li MC, Li X,Lim SS, Lin J, Liu X, Lohner V, Lorenzovici L, Lotufo PA, Lucchetti G, Lusk JB, Lutzky Saute R, M Amin HI, Malhotra AK, Malhotra K, Malik AA, Malta DC, Mansournia MA, Mantovani LG, Manu E, Marateb HR, Marino M, Maroufi SF, Martinez-Piedra R, Martini S, Martorell M, Marzo RR, Mathangasinghe Y, Mathews E, Maugeri A, McPhail SM, Mehmood A, Mehndiratta MM, Mehrabani-Zeinabad K, Menezes RG, Meo SA, Meretoja A, Mestrovic T, Mettananda CDK, Miazgowski T, Micheletti Gomide Nogueira de Sá AC, Minervini G, Minh LHN, Mirica A, Mirrakhimov EM, Mirza-Aghazadeh-Attari M, Mishra AK, Mithra P, Mohamed AZ, Mohamed AI, Mohammad AM, Mohammadi S, Mohammadian-Hafshejani A, Mohammed S, Mokdad AH, Molinaro S, Momani S, Moni MA, Moodi Ghalibaf A, Moradi M, Moradi Y, Moraga P, Morawska L,Msherghi A, Munjal K, Murray CJL, Nagarajan AJ, Naik GR, Najdaghi S, Nakhostin Ansari N, Nargus S, Davani DN, Natto ZS, Nauman J, Nayak VC, Nazri-Panjaki A, Negoi RI, Nematollahi S, Newton CRJ, Nguyen DH, Nguyen HTH, Nguyen HQ, Nguyen PT, Nguyen VT, Niazi RK, Nigatu YT, Nikoobar A, Nogueira de Sá AT, Nomura S, Noubiap JJ, Nugen F, Nzoputam CI, Oancea B, Oduro MS, Ojo-Akosile TR, Okati-Aliabad H, Okeke SR, Okekunle AP, Olagunju AT,Olaiya MT, Oliveira AB, Oliveira GMM, Olorukooba AA, Olufadewa II, Ornello R, Ortiz-Prado E, Osuagwu UL, Ouyahia A, Owolabi MO, Ozair A, P A MP, Padron-Monedero A, Padubidri JR, Panagiotakos D, Panos GD, Panos LD, Pantazopoulos I, Parikh RR, Park S, Patel J, Patel UK, Patoulias D, Pedersini P, Peprah EK, Pereira G, Perianayagam A, Perico N, Perna S, Petermann-Rocha FE, Philip AK, Piradov MA, Plotnikov E, Polibin RV, Postma MJ, Pradhan J, Prasad M, Puvvula J, Qasim NH, Qian G, Raggi A, Rahim F, Rahimi-Movaghar V, Rahman M, Rahman MA, Rahmani AM, Rahmanian M, Rajaa S, Rajabpour Sanati A, Rajpoot PL, Rajput P, Ramadan MM, Ramasamy SK, Ramazanu S, Rane A, Rashedi S, Rashidi MM,Rathish D, Rawaf S, Razo C, Reddy MMRK, Redwan E, Remuzzi G, Rezaei N, Rezaei N, Rezaeian M, Rocha HAL, Rodriguez JAB, Roever L, Romoli M, Romozzi M, Ross AG, Rout HS, Roy N, Roy P, Saad AMA, Saadatian Z, Sabour S, Sacco S, Saddik BA, Sadeghi E, Saeed U,Saheb Sharif-Askari F, Sahebkar A, Sahoo PM, Sajib MRUZ, Salaroli LB, Saleh MA, Samodra YL, Samuel VP, Samy AM, Santric-Milicevic MM, Saravanan A, Sarkar T, Sarode GS, Sarode SC, Sartorius B, Satpathy M, Schlaich MP, Schneider IJC, Schuermans A, Selvaraj S,Senthilkumaran S, Sepanlou SG, Sethi Y, Seylani A, Shaaban AN, Shafie M, Shahwan MJ,Shaikh MA, Shaikh SZ, Shamim MA, Shamsi A, Shamsutdinova A, Shanawaz M, Shannawaz M, Sharifan A, Sharifi Rad J, Sharma V, Shashamo BB, Shetty M, Shetty PK, Shigematsu M, Shittu A, Shiue I, Shlobin NA, Shorofi SA, Siddig EE, Singh B, Singh P, Singh P,Singh S, Sobia F, Solanki R, Solanki S, Soraneh S, Spartalis M, Srinivasamurthy SK,Stanaway JD, Stanikzai MH, Starodubova AV, Sun J, Sun Z, Swain CK, Szarpak L, Tabaee Damavandi P, Tabatabaei SM, Tabatabaeizadeh SA, Tabche C, Taiba J, Talaat IM, Tamuzi JL, Tan KK, Temsah MH, Teramoto M, Thakur R, Thankappan KR, Thayakaran R, Thirunavukkarasu S, Ticoalu JHV, Tiwari K, Tonelli M, Topor-Madry R, Tovani-Palone MR, Tran AT, Tran JT, Tran TH, Tran Minh Duc N, Truelsen TC, Truyen TTTT, Tsai DH, Ullah A, Unim B,Unnikrishnan B, Unsworth CA, Usman JS, Vahdati S, Vaithinathan AG, Valizadeh R, Van den Eynde J, Varghese J, Vasankari TJ, Venketasubramanian N, Vervoort D, Villafañe JH, Vinayak M, Vladimirov SK, Wafa HA, Waheed Y, Wahood W, Walde MT, Wang Y, Wickramasinghe ND, Willeit P, Wolde AA, Wolfe CDA, Wubie YM, Xiao H, Xu S, Xu X, Yamagishi K, Yano Y, Yarahmadi A, Yaribeygi H, Yaya S, Ye P, Yon DK, Yonemoto N, Yu C, Zanghì A, Zare I, Zastrozhin M, Zhang C, Zhang Y, Zhang ZJ, Zhang Z, Zhao H, Zhou SC, Zhumagaliuly A, Zia H, Zielinska M, Zyoud SH, Roth GA, Feigin VL. Global, Regional, and National Burden of Nontraumatic Subarachnoid Hemorrhage: The Global Burden of Disease Study 2021. JAMA Neurol. 2025;82(8):765–87. 10.1001/jamaneurol.2025.1522 PMC12557468 40406922

[36] Ronkainen A, Niskanen M, Rinne J, Koivisto T, Hernesniemi J, Vapalahti M. Evidence for Excess Long-Term Mortality After Treated Subarachnoid Hemorrhage. Stroke. 2001;32:2850–3. 10.1161/hs1201.099711. 11739986

[37] Cesarini KG, Hårdemark HG, Persson L. Improved survival after aneurysmal subarachnoid hemorrhage: review of case management during a 12-year period. J Neurosurg. 1999;90(4):664–72. 10.3171/jns.1999.90.4.0664. 10193611

[38] Haug T, Sorteberg A, Finset A, Lindegaard KF, Lundar T, Sorteberg W. Cognitive functioning and health-related quality of life 1 year after aneurysmal subarachnoid hemorrhage in preoperative comatose patients (Hunt and Hess Grade V patients). Neurosurgery. 2010;66(3):475–84. 10.1227/01.NEU.0000365364.87303.AC. discussion 484-5. 20124932

[39] Capion T, Lilja-Cyron A, Olsen MH, Møller K, Juhler M, Mathiesen T. Predictors of shunt insertion in patients with aneurysmal subarachnoid haemorrhage-a single-centre retrospective analysis. Acta Neurochir (Wien). 2024;166(1):24. 10.1007/s00701-024-05926-1. PMC10799140 38240848

[40] Mayer SA, Bruder N, Citerio G, Defreyne L, Dubois C, Gupta R, Higashida R, Marr A, Nguyen TN, Roux S, Smrčka M, Torné RT, Aldrich EF. on behalf of the REACT investigators. REACT: a randomized trial to assess the efficacy and safety of clazosentan for preventing clinical deterioration due to delayed cerebral ischemia after aneurysmal subarachnoid hemorrhage. J Neurosurg. 2024;142(1):98–109. 10.3171/2024.4.JNS232191. 39126720

[41] Macdonald RL, Higashida RT, Keller E, Mayer SA, Molyneux A, Raabe A, Vajkoczy P, Wanke I, Bach D, Frey A, Nowbakht P, Roux S, Kassell N. Randomized trial of clazosentan in patients with aneurysmal subarachnoid hemorrhage undergoing. 10.1161/STROKEAHA.111.648980 22403047

[42] Lauzier DC, et al. Early Brain Injury After Subarachnoid Hemorrhage: Incidence and Mechanisms. Stroke. 2023;54:1426–40. 10.1161/STROKEAHA.122.040072. PMC10243167 36866673

[43] Ansar S, Maddahi A, Edvinsson L. Inhibition of cerebrovascular raf activation attenuates cerebral blood flow and prevents upregulation of contractile receptors after subarachnoid hemorrhage. BMC Neurosci. 2011;12:107. 10.1186/1471-2202-12-107. PMC3219635 22032648

[44] Christensen ST, et al. Pre-clinical effects of highly potent MEK1/2 inhibitors on rat cerebral vasculature after organ culture and subarachnoid haemorrhage. Clin Sci (Lond). 2019;133:1797–811. 10.1042/CS20190636. 31387935

[45] Muller AH, et al. Proteomic Expression Changes in Large Cerebral Arteries After Experimental Subarachnoid Hemorrhage in Rat Are Regulated by the MEK-ERK1/2 Pathway. J Mol Neurosci. 2017;62:380–94. 10.1007/s12031-017-0944-7. PMC5541124 28741142

[46] Povlsen GK, Johansson SE, Larsen CC, Samraj AK, Edvinsson L. Early events triggering delayed vasoconstrictor receptor upregulation and cerebral ischemia after subarachnoid hemorrhage. BMC Neurosci. 2013;14:34. 10.1186/1471-2202-14-34. PMC3618233 23496889

