# Supplementary material for: First-in-Human Phase 1b/2a Trial Shows Safety with MEK 1/2 Inhibitor EDV2209 as A Novel Treatment for Spontaneous Subarachnoid Hemorrhage
Source: Transl Stroke Res. 2026 Oct 30;17(6):116. doi: 10.1007/s12975-026-01504-y (PMC13719763; doi:10.1007/s12975-026-01504-y)
Supplement: Supplementary file 1 [file 12975_2026_1504_MOESM1_ESM.docx]

Supplement 1. Animal toxicity studies

Investigation of the cardiovascular and respiratory function in conscious but restrained dogs two hours after the first dose in a pivotal 8-day repeat ICV dose study revealed no untoward effects on the 12-lead ECG, blood pressure, respiratory rate or lung sounds at any dose level. The highest dose investigated was 7-fold the predicted ventricular Cmax in humans at the proposed clinical dose. EDV2209 shows limited passage over the blood-CSF barrier; accordingly, the ICV-route of administration causes a systemic exposure in plasma which is many orders of magnitudes lower than what is required to obtain pharmacological effect via the intravenous route of administration (see Section 1.5.1).

By use of a functional observation battery, rats included in a repeat-dose toxicity study were tested before the start of the study and then again on day 6 for CNS toxicity using standard methods. Three high-dose female rats administered EDV2209 at an ICV dose that was 14-fold the predicted ventricular Cmax in humans showed signs of neurological impairment (head tilt and circling movements). The NOAEL (No-Observed-Adverse-Effect Level) in rats was 7-fold the predicted ventricular Cmax in humans.

Dogs included in an 8-day repeat-dose toxicity study were also subjected to a faecal occult blood testing. This was done prior to start of treatment and after the first and last dose. Normal behaviors were generally observed prior to dosing and 90 minutes after the first dose. After the last dose, 14 of 21 animals showed one or more abnormal behavior/symptom (abnormal body position, slight palpebral closure, twitching of the head, reduced vigorous movement, change in gait). The routine observations throughout the study also showed presence of CNS symptoms (lethargy, shaking body or head/jaw and/or change in body posture), beginning already after the first dosing occasion. Since all aforementioned signs of CNS toxicity were observed across all treatment groups, including the two control groups (vehicle control and aCSF control), these were judged to be procedure rather than treatment related. Moreover, dogs receiving aCSF with or without the excipient Kolliphor® EL were also equally affected, thus supporting that Kolliphor® EL at exposures equivalent to projected human ICV exposures had no untoward effects.

Histopathological examination of rats and dogs in repeat-dose toxicity studies displayed similar findings with signs of inflammation in the brain that were similar in all groups. There was an association of severity and incidence of findings with proximity to the injection site. Again, this was judged to be procedure rather than treatment related. There were no other histopathological findings in any other organ.

To summarize, pivotal repeat-dose toxicity studies have been carried out in rats and dogs and EDV2209 has also undergone in vitro genotoxicity testing. Toxicity to reproduction has not been investigated. Overall, the non-clinical safety investigations of EDV2209 have established that:

• The NOAEL in the repeat-dose ICV study in rats is 7-fold as high as the predicted ICV Cmax in humans with the selected clinical dose. The CNS toxicity occurred in rats at 14-fold the predicted human ventricular Cmax.

• There was no obvious treatment-related toxicity in dogs administered repeat ICV doses for 8 days at 7-fold the predicted human ventricular Cmax. The CNS symptoms were recorded following the first injection and throughout the 8-day dosing period in dogs without any dose-effect relationship and were judged to be procedure-related rather than related to the test compound.

• Histopathology examination only showed findings in the brains of rats and dogs that were judged to be procedure related.

• No relevant in vitro genotoxic activity was seen when EDV2209 was tested in Ames Salmonella Assay and in a chromosome aberration assay using human lymphocytes.

Supplement 2. Vital signs

Systolic blood pressure

| Systolic blood pressure (mmHg): Dose 1 | | | | |
| --- | --- | --- | --- | --- |
| Group | Statistics | Pre Dose 1 | 1 h Post Dose 1 | Post Dose 1 - Pre dose 1 |
| Cohort 4+5 | N | 13 | 13 | 13 |
|  |  |  |  |  |
|  | Min | 87 | 105 | -51 |
|  | Median | 121.00 | 138.00 | 5.00 |
|  | Max | 183 | 161 | 71 |
|  | Mean | 122.08 | 132.62 | 10.54 |
|  | Std | 27.02 | 17.45 | 30.22 |
|  |  |  |  |  |
|  | P-value^[1]^ | NA | NA | 0.0979 |
| Placebo | N | 5 | 5 | 5 |
|  | Min | 106 | 96 | -24 |
|  | Median | 119.00 | 108.00 | -9.00 |
|  | Max | 143 | 134 | 0 |
|  | Mean | 120.00 | 110.80 | -9.20 |
|  | Std | 14.05 | 15.99 | 9.09 |
|  |  |  |  |  |
|  | P-value^1^ | NA | NA | 0.1250 |
| P-value^[2]^ |  | 0.8653 | 0.0287 | 0.0186 |

| Systolic blood pressure: Dose 2. | | | | |
| --- | --- | --- | --- | --- |
| Group | Statistics | Pre Dose 2 | 1 h Post Dose 2 | Post Dose 2 - Pre dose 2 |
| Cohort 4+5 | N | 13 | 13 | 13 |
|  |  |  |  |  |
|  | Min | 101 | 96 | -44 |
|  | Median | 122.00 | 115.00 | -4.00 |
|  | Max | 159 | 144 | 5 |
|  | Mean | 126.00 | 118.00 | -8.00 |
|  | Std | 17.65 | 15.30 | 12.92 |
|  |  |  |  |  |
|  | P-value^1^ | NA | NA | 0.0391 |
| Placebo | N | 3 | 3 | 3 |
|  | Missing | 2 | 2 | 2 |
|  | Min | 119 | 107 | -12 |
|  | Median | 123.00 | 122.00 | -1.00 |
|  | Max | 127 | 134 | 7 |
|  | Mean | 123.00 | 121.00 | -2.00 |
|  | Std | 4.00 | 13.53 | 9.54 |
|  |  |  |  |  |
|  | P-value^1^ | NA | NA | 0.7500 |
| P-value^2^ |  | 0.9857 | 0.6893 | 0.4125 |

| Systolic blood pressure: Dose 3. | | | | |
| --- | --- | --- | --- | --- |
| Group | Statistics | Pre Dose 3 | 1 h Post Dose 3 | Post Dose 3 - Pre dose 3 |
| Cohort 4+5 | N | 13 | 13 | 13 |
|  |  |  |  |  |
|  | Min | 87 | 104 | -42 |
|  | Median | 120.00 | 124.00 | 5.00 |
|  | Max | 184 | 164 | 30 |
|  | Mean | 124.77 | 129.54 | 4.77 |
|  | Std | 24.99 | 21.57 | 18.54 |
|  |  |  |  |  |
|  | P-value^1^ | NA | NA | 0.3159 |
| Placebo | N | 3 | 3 | 3 |
|  |  |  |  |  |
|  | Min | 121 | 118 | -3 |
|  | Median | 121.00 | 136.00 | 15.00 |
|  | Max | 122 | 138 | 16 |
|  | Mean | 121.33 | 130.67 | 9.33 |
|  | Std | 0.58 | 11.02 | 10.69 |
|  |  |  |  |  |
|  | P-value^1^ | NA | NA | 0.5000 |
| P-value^2^ |  | 0.8786 | 1.0000 | 0.8821 |

Diastolic blood pressure

| Diastolic blood pressure (mmHg): Dose 1. | | | | |
| --- | --- | --- | --- | --- |
| Group | Statistics | Pre Dose 1 | 1 h Post Dose 1 | Post Dose 1 - Pre dose 1 |
| Cohort 4+5 | N | 13 | 13 | 13 |
|  |  |  |  |  |
|  | Min | 50 | 54 | -28 |
|  | Median | 61.00 | 72.00 | 3.00 |
|  | Max | 96 | 84 | 29 |
|  | Mean | 65.15 | 69.15 | 4.00 |
|  | Std | 13.70 | 9.74 | 13.79 |
|  |  |  |  |  |
|  | P-value^1^ | NA | NA | 0.1240 |
| Placebo | N | 5 | 5 | 5 |
|  |  |  |  |  |
|  | Min | 53 | 45 | -18 |
|  | Median | 57.00 | 48.00 | -10.00 |
|  | Max | 96 | 78 | -5 |
|  | Mean | 65.40 | 54.00 | -11.40 |
|  | Std | 17.56 | 13.66 | 5.46 |
|  |  |  |  |  |
|  | P-value^1^ | NA | NA | 0.0625 |
| P-value^2^ |  | 0.9412 | 0.0208 | 0.0039 |

| Diastolic blood pressure: Dose 2. | | | | |
| --- | --- | --- | --- | --- |
| Group | Statistics | Pre Dose 2 | 1 h Post Dose 2 | Post Dose 2 - Pre dose 2 |
| Cohort 4+5 | N | 13 | 13 | 13 |
|  |  |  |  |  |
|  | Min | 45 | 43 | -9 |
|  | Median | 62.00 | 54.00 | -4.00 |
|  | Max | 79 | 73 | 4 |
|  | Mean | 60.08 | 56.31 | -3.77 |
|  | Std | 10.32 | 8.24 | 3.88 |
|  |  |  |  |  |
|  | P-value^1^ | NA | NA | 0.0068 |
| Placebo | N | 3 | 3 | 3 |
|  |  |  |  |  |
|  | Min | 48 | 47 | -9 |
|  | Median | 58.00 | 52.00 | -4.00 |
|  | Max | 61 | 54 | -1 |
|  | Mean | 55.67 | 51.00 | -4.67 |
|  | Std | 6.81 | 3.61 | 4.04 |
|  |  |  |  |  |
|  | P-value^1^ | NA | NA | 0.2500 |
| P-value^2^ |  | 0.4982 | 0.3821 | 0.8821 |

| Diastolic blood pressure: Dose 3. | | | | |
| --- | --- | --- | --- | --- |
| Group | Statistics | Pre Dose 3 | 1 h Post Dose 3 | Post Dose 3 - Pre dose 3 |
| Cohort 4+5 | N | 13 | 13 | 13 |
|  |  |  |  |  |
|  | Min | 49 | 47 | -6 |
|  | Median | 61.00 | 68.00 | 1.00 |
|  | Max | 73 | 91 | 27 |
|  | Mean | 61.31 | 67.85 | 6.54 |
|  | Std | 9.04 | 12.44 | 11.44 |
|  |  |  |  |  |
|  | P-value^1^ | NA | NA | 0.1326 |
| Placebo | N | 3 | 3 | 3 |
|  |  |  |  |  |
|  | Min | 47 | 45 | -2 |
|  | Median | 49.00 | 53.00 | 4.00 |
|  | Max | 58 | 67 | 9 |
|  | Mean | 51.33 | 55.00 | 3.67 |
|  | Std | 5.86 | 11.14 | 5.51 |
|  |  |  |  |  |
|  | P-value^1^ | NA | NA | 0.5000 |
| P-value^2^ |  | 0.0750 | 0.1375 | 1.0000 |

Pulse

| Pulse (Beats / minute): Dose 1. | | | | |
| --- | --- | --- | --- | --- |
| Group | Statistics | Pre Dose 1 | 1 h Post Dose 1 | Post Dose 1 - Pre dose 1 |
| Cohort 4+5 | N | 13 | 13 | 13 |
|  |  |  |  |  |
|  | Min | 46 | 44 | -13 |
|  | Median | 69.00 | 63.00 | -2.00 |
|  | Max | 106 | 101 | 25 |
|  | Mean | 68.15 | 71.00 | 2.85 |
|  | Std | 17.39 | 18.89 | 10.33 |
|  |  |  |  |  |
|  | P-value^1^ | NA | NA | 0.4885 |
| Placebo | N | 5 | 5 | 5 |
|  |  |  |  |  |
|  | Min | 48 | 48 | -8 |
|  | Median | 69.00 | 61.00 | 0.00 |
|  | Max | 76 | 82 | 6 |
|  | Mean | 64.00 | 63.60 | -0.40 |
|  | Std | 11.29 | 14.60 | 6.11 |
|  |  |  |  |  |
|  | P-value^1^ | NA | NA | 1.0000 |
| P-value^2^ |  | 0.9376 | 0.4863 | 0.6192 |

| Pulse: Dose 2. | | | | |
| --- | --- | --- | --- | --- |
| Group | Statistics | Pre Dose 2 | 1 h Post Dose 2 | Post Dose 2 - Pre dose 2 |
| Cohort 4+5 | N | 13 | 13 | 13 |
|  |  |  |  |  |
|  | Min | 42 | 42 | -16 |
|  | Median | 64.00 | 62.00 | 0.00 |
|  | Max | 92 | 93 | 11 |
|  | Mean | 69.31 | 68.46 | -0.85 |
|  | Std | 18.23 | 16.09 | 6.90 |
|  |  |  |  |  |
|  | P-value^1^ | NA | NA | 0.7422 |
| Placebo | N | 3 | 3 | 3 |
|  |  |  |  |  |
|  | Min | 55 | 52 | -8 |
|  | Median | 62.00 | 54.00 | -3.00 |
|  | Max | 67 | 68 | 1 |
|  | Mean | 61.33 | 58.00 | -3.33 |
|  | Std | 6.03 | 8.72 | 4.51 |
|  |  |  |  |  |
|  | P-value^1^ | NA | NA | 0.5000 |
| P-value^2^ |  | 0.7696 | 0.1946 | 0.5929 |

| Pulse: Dose 3. | | | | |
| --- | --- | --- | --- | --- |
| Group | Statistics | Pre Dose 3 | 1 h Post Dose 3 | Post Dose 3 - Pre dose 3 |
| Cohort 4+5 | N | 13 | 13 | 13 |
|  |  |  |  |  |
|  | Min | 46 | 47 | -13 |
|  | Median | 67.00 | 71.00 | -2.00 |
|  | Max | 127 | 124 | 9 |
|  | Mean | 72.08 | 70.92 | -1.15 |
|  | Std | 20.78 | 21.45 | 6.83 |
|  |  |  |  |  |
|  | P-value^1^ | NA | NA | 0.6238 |
| Placebo | N | 3 | 3 | 3 |
|  |  |  |  |  |
|  | Min | 62 | 59 | -4 |
|  | Median | 66.00 | 67.00 | -3.00 |
|  | Max | 71 | 72 | 6 |
|  | Mean | 66.33 | 66.00 | -0.33 |
|  | Std | 4.51 | 6.56 | 5.51 |
|  |  |  |  |  |
|  | P-value^1^ | NA | NA | 1.0000 |
| P-value^2^ |  | 0.8839 | 0.7196 | 1.0000 |

Temperature

| Temperature (^o^C): Dose 1. | | | | |
| --- | --- | --- | --- | --- |
| Group | Statistics | Pre Dose 1 | 1 h Post Dose 1 | Post Dose 1 - Pre dose 1 |
| Cohort 4+5 | N | 12 | 12 | 11 |
|  |  |  |  |  |
|  | Min | 33.9 | 34.6 | -0.5 |
|  | Median | 35.60 | 36.00 | 0.50 |
|  | Max | 37.1 | 37.5 | 0.7 |
|  | Mean | 35.63 | 35.97 | 0.28 |
|  | Std | 1.04 | 0.93 | 0.40 |
|  |  |  |  |  |
|  | P-value^1^ | NA | NA | 0.0361 |
| Placebo | N | 5 | 5 | 5 |
|  |  |  |  |  |
|  | Min | 35 | 34.9 | -0.3 |
|  | Median | 35.40 | 35.40 | 0.20 |
|  | Max | 36.2 | 37.9 | 1.7 |
|  | Mean | 35.52 | 35.90 | 0.38 |
|  | Std | 0.48 | 1.19 | 0.79 |
|  |  |  |  |  |
|  | P-value^1^ | NA | NA | 0.4375 |
| P-value^2^ |  | 0.7797 | 0.9000 | 0.7257 |

| Temperature: Dose 2. | | | | |
| --- | --- | --- | --- | --- |
| Group | Statistics | Pre Dose 2 | 1 h Post Dose 2 | Post Dose 2 - Pre dose 2 |
| Cohort 4+5 | N | 13 | 13 | 13 |
|  |  |  |  |  |
|  | Min | 35.1 | 35.2 | -0.4 |
|  | Median | 36.80 | 37.20 | 0.10 |
|  | Max | 38.5 | 38.2 | 0.5 |
|  | Mean | 36.88 | 37.03 | 0.15 |
|  | Std | 0.99 | 0.89 | 0.29 |
|  |  |  |  |  |
|  | P-value^1^ | NA | NA | 0.0840 |
| Placebo | N | 3 | 3 | 3 |
|  |  |  |  |  |
|  | Min | 36 | 36.3 | -0.1 |
|  | Median | 36.40 | 36.40 | 0.30 |
|  | Max | 37.1 | 37.4 | 0.4 |
|  | Mean | 36.50 | 36.70 | 0.20 |
|  | Std | 0.56 | 0.61 | 0.26 |
|  |  |  |  |  |
|  | P-value^1^ | NA | NA | 0.5000 |
| P-value^2^ |  | 0.4607 | 0.5982 | 1.0000 |

| Temperature: Dose 3. | | | | |
| --- | --- | --- | --- | --- |
| Group | Statistics | Pre Dose 3 | 1 h Post Dose 3 | Post Dose 3 - Pre dose 3 |
| Cohort 4+5 | N | 13 | 13 | 13 |
|  |  |  |  |  |
|  | Min | 35.4 | 35.5 | -0.4 |
|  | Median | 37.00 | 36.90 | 0.10 |
|  | Max | 38 | 38 | 0.3 |
|  | Mean | 36.92 | 36.93 | 0.02 |
|  | Std | 0.85 | 0.79 | 0.22 |
|  |  |  |  |  |
|  | P-value^1^ | NA | NA | 0.6299 |
| Placebo | N | 3 | 3 | 3 |
|  |  |  |  |  |
|  | Min | 37 | 36.9 | -0.2 |
|  | Median | 37.10 | 37.00 | 0.00 |
|  | Max | 37.6 | 37.7 | 0.1 |
|  | Mean | 37.23 | 37.20 | -0.03 |
|  | Std | 0.32 | 0.44 | 0.15 |
|  |  |  |  |  |
|  | P-value^1^ | NA | NA | 1.0000 |
| P-value^2^ |  | 0.6786 | 0.5875 | 0.5518 |

RESP

| Respiration (frequence/minute): Dose 1. | | | | |
| --- | --- | --- | --- | --- |
| Group | Statistics | Pre Dose 1 | 1 h Post Dose 1 | Post Dose 1 - Pre dose 1 |
| Cohort 4+5 | N | 13 | 13 | 13 |
|  |  |  |  |  |
|  | Min | 11 | 8 | -5 |
|  | Median | 14.00 | 14.00 | 0.00 |
|  | Max | 19 | 19 | 5 |
|  | Mean | 13.69 | 13.85 | 0.15 |
|  | Std | 2.02 | 2.67 | 2.41 |
|  |  |  |  |  |
|  | P-value^1^ | NA | NA | 0.8438 |
| Placebo | N | 5 | 5 | 5 |
|  |  |  |  |  |
|  | Min | 12 | 10 | -6 |
|  | Median | 14.00 | 12.00 | -2.00 |
|  | Max | 18 | 14 | 0 |
|  | Mean | 14.20 | 11.80 | -2.40 |
|  | Std | 2.28 | 1.48 | 2.61 |
|  |  |  |  |  |
|  | P-value^1^ | NA | NA | 0.2500 |
| P-value^2^ |  | 0.7835 | 0.0638 | 0.0893 |

| Respiration: Dose 2. | | | | |
| --- | --- | --- | --- | --- |
| Group | Statistics | Pre Dose 2 | 1 h Post Dose 2 | Post Dose 2 - Pre dose 2 |
| Cohort 4+5 | N | 13 | 13 | 13 |
|  |  |  |  |  |
|  | Min | 8 | 9 | -5 |
|  | Median | 14.00 | 14.00 | 0.00 |
|  | Max | 20 | 20 | 4 |
|  | Mean | 14.38 | 14.23 | -0.15 |
|  | Std | 3.88 | 3.72 | 2.51 |
|  |  |  |  |  |
|  | P-value^1^ | NA | NA | 0.9141 |
| Placebo | N | 3 | 3 | 3 |
|  |  |  |  |  |
|  | Min | 10 | 10 | -2 |
|  | Median | 12.00 | 12.00 | 0.00 |
|  | Max | 16 | 14 | 0 |
|  | Mean | 12.67 | 12.00 | -0.67 |
|  | Std | 3.06 | 2.00 | 1.15 |
|  |  |  |  |  |
|  | P-value^1^ | NA | NA | 1.0000 |
| P-value^2^ |  | 0.5714 | 0.4304 | 0.4857 |

| Respiration: Dose 3. | | | | |
| --- | --- | --- | --- | --- |
| Group | Statistica | Pre Dose 3 | 1 h Post Dose 3 | Post Dose 3 - Pre dose 3 |
| Cohort 4+5 | N | 13 | 13 | 13 |
|  |  |  |  |  |
|  | Min | 10 | 10 | -6 |
|  | Median | 15.00 | 17.00 | 0.00 |
|  | Max | 24 | 24 | 9 |
|  | Mean | 15.62 | 16.77 | 1.15 |
|  | Std | 4.50 | 4.69 | 3.48 |
|  |  |  |  |  |
|  | P-value^1^ | NA | NA | 0.3125 |
| Placebo | N | 3 | 3 | 3 |
|  |  |  |  |  |
|  | Min | 12 | 12 | 0 |
|  | Median | 12.00 | 13.00 | 0.00 |
|  | Max | 14 | 14 | 1 |
|  | Mean | 12.67 | 13.00 | 0.33 |
|  | Std | 1.15 | 1.00 | 0.58 |
|  |  |  |  |  |
|  | P-value^1^ | NA | NA | 1.0000 |
| P-value^2^ |  | 0.3571 | 0.3161 | 0.7982 |

Supplement 3. Laboratory tests

| **Clinical Chemistry**   - Bilirubin - Aspartate aminotransferase (ASAT) - Alanine aminotransferase (ALAT) - Glutamyl transpeptidase (GT) - Alkaline phosphatase (ALP) - C-reactive protein (CRP) - Amylase - Albumin - Creatinine - Glucose - Potassium - Sodium - Chloride - Calcium | **Hematology**   - Hemoglobin (Hb) - White Blood Cell Count - Platelet count - Prothrombin complex, International Normalized Ratio (INR) - Activated partial thromboplastin time (APTT) | **Arterial Blood Gas**   - pH - pCO_2_ - pO_2_ - Base excess (BE) - Lactate |
| --- | --- | --- |

Supplement 4 Adverse events per patient

| Adverse event | Number of patients with at least one report | | |
| --- | --- | --- | --- |
|  | Placebo (N=5) | EDV2209 (N=22) | Total |
|  |  |  |  |
| **Infections and infestations** |  |  |  |
| Urinary tract infection | 4 | 11 | 15 |
| Pulmonary infection | 0 | 10 | 10 |
| Sepsis | 2 | 1 | 3 |
| Ventriculitis | 1 | 5 | 6 |
| Skin infection | 1 | 1 | 2 |
| Thrush | 1 | 5 | 6 |
| Device related infection | 0 | 2 | 2 |
| **Psychiatric disorders** |  |  |  |
| Depression | 1 | 1 | 2 |
| Hallucinations | 0 | 1 | 1 |
| Insomnia | 2 | 15 | 17 |
| Other: Withdrawal syndrome | 0 | 4 | 4 |
| **Nervous system disorders** |  |  |  |
| Depressed level of consciousness | 4 | 11 | 15 |
| Cerebral edema | 1 | 4 | 5 |
| Cerebral haemorrhage | 1 | 3 | 4 |
| Cerebral infarction | 1 | 7 | 8 |
| Cognitive disorder | 3 | 4 | 7 |
| Delayed ischaemic neurological deficit | 0 | 2 | 2 |
| Delirium | 2 | 5 | 7 |
| Epilepsy | 0 | 1 | 1 |
| Headache | 4 | 11 | 15 |
| Hydrocephalus | 1 | 6 | 7 |
| Intracranial pressure increased | 3 | 5 | 8 |
| Subdural hygroma | 1 | 0 | 1 |
| Transient ischaemic attack | 0 | 1 | 1 |
| IIIrd nerve paralysis | 0 | 1 | 1 |
| VIth nerve paralysis | 0 | 1 | 1 |
| Pneumocephalus | 0 | 2 | 2 |
| Dizziness | 3 | 7 | 10 |
| Aneurysm reruptured | 0 | 1 | 1 |
| **Eye disorders** |  |  |  |
| Eye irritation | 0 | 1 | 1 |
| Dry eye | 0 | 2 | 2 |
| Vitreous haemorrhage | 0 | 2 | 2 |
| Blurred vision | 2 | 3 | 5 |
| Periorbital oedema | 0 | 2 | 2 |
| **Ear and labyrinth disorders** |  |  |  |
| Hyperacusis | 1 | 2 | 3 |
| Tinnitus | 0 | 1 | 1 |
| **Cardiovascular disorders** |  |  |  |
| Atrial fibrillation | 1 | 2 | 3 |
| Bradycardia | 1 | 4 | 5 |
| Tachycardia | 2 | 5 | 7 |
| Stress cardiomyopathy | 0 | 1 | 1 |
| Other arythmia | 1 | 5 | 6 |
| Phlebitis | 1 | 0 | 1 |
| Peripheral pseudoaneurysm | 0 | 1 | 1 |
| Hypertension | 4 | 7 | 11 |
| Hypotension | 4 | 7 | 11 |
| Hypovolemia | 2 | 1 | 3 |
| ECG: QT prolonged | 2 | 8 | 10 |
| ECG ST depression | 1 | 2 | 3 |
| ECG T wave abnormal | 0 | 3 | 3 |
| **Respiratory, thoracic and mediastinal disorders** |  |  |  |
| Acute respiratory distress syndrome | 0 | 1 | 1 |
| Bronchospasm | 3 | 2 | 5 |
| Dyspnea | 0 | 1 | 1 |
| Hypoxia | 1 | 2 | 3 |
| Pleural effusion | 0 | 2 | 2 |
| Pulmonary oedema | 1 | 0 | 1 |
| Tracheal deviation | 1 | 0 | 1 |
| Epistaxis | 0 | 1 | 1 |
| Other: Angioedema | 1 | 0 | 1 |
| **Gastrointestinal disorders** |  |  |  |
| Abdominal pain | 1 | 4 | 5 |
| Constipation | 5 | 18 | 23 |
| Impaired gastric emptying | 1 | 3 | 4 |
| Nausea | 2 | 10 | 12 |
| Stress ulcer | 0 | 1 | 1 |
| Vomiting | 3 | 3 | 6 |
| Diarrhoea | 1 | 0 | 1 |
| Haemorrhoids | 0 | 2 | 2 |
| Dysphagia | 3 | 3 | 6 |
| **Skin and subcutaneous tissue disorders** |  |  |  |
| Decubitus ulcer | 1 | 2 | 3 |
| Eczema | 1 | 1 | 2 |
| Erythema | 0 | 1 | 1 |
| Miliaria | 0 | 1 | 1 |
| Alopecia | 0 | 1 | 1 |
| **Musculoskeletal and connective tissue disorders** |  |  |  |
| Neck pain | 2 | 10 | 12 |
| Myopathy | 1 | 1 | 2 |
| Myalgia | 2 | 5 | 7 |
| Back pain | 0 | 7 | 7 |
| Arthralgia | 0 | 1 | 1 |
| **Renal and urinary disorders** |  |  |  |
| Urinary retention | 1 | 3 | 4 |
| Haematuria | 1 | 0 | 1 |
| **Reproductive system disorders** |  |  |  |
| Vaginal discharge | 1 | 0 | 1 |
| Vaginal haemorrhage | 0 | 1 | 1 |
| Cervicitis | 0 | 1 | 1 |
| Epididymitis | 0 | 1 | 1 |
| Oedema genital | 1 | 0 | 1 |
| **General disorders and administration site conditions** |  |  |  |
| Edema peripheral | 2 | 10 | 12 |
| Critical illness | 0 | 1 | 1 |
| Infusion site extravasation | 1 | 1 | 2 |
| Diabetes insipidus | 0 | 1 | 1 |
| **Blood biomarkers** |  |  |  |
| Gamma-glutamyltransferase increased | 4 | 13 | 17 |
| Blood alkaline phosphatase increased | 2 | 6 | 8 |
| Blood albumin decreased | 4 | 16 | 20 |
| Aspartate aminotransferase increased | 3 | 12 | 15 |
| Alanine aminotransferase increased | 3 | 11 | 14 |
| Amylase increased | 1 | 4 | 5 |
| Blood lactate dehydrogenase increased | 2 | 4 | 6 |
| Blood leukocytes increased | 0 | 2 | 2 |
| Blood platelet count increased | 2 | 7 | 9 |
| Blood hemoglobin decreased | 4 | 13 | 17 |
|  |  |  |  |
| *Electrolytes and trace elements* |  |  |  |
| Blood zinc decreased | 3 | 9 | 12 |
| Blood sodium decreased | 1 | 12 | 13 |
| Blood sodium increased | 1 | 3 | 4 |
| Blood potassium decreased | 4 | 13 | 17 |
| Blood potassium increased | 1 | 0 | 1 |
| Blood phosphate decreased | 2 | 12 | 14 |
| Blood phosphate increased | 0 | 1 | 1 |
| Blood magnesium increased | 0 | 1 | 1 |
| Blood magnesium decreased | 0 | 1 | 1 |
| Blood chloride increased | 0 | 2 | 2 |
| Blood calcium decreased | 2 | 5 | 7 |
| Acidosis | 2 | 4 | 6 |
| Blood creatinine increased | 1 | 0 | 1 |
| C-reactive protein increased | 2 | 3 | 5 |
| Blood glucose increased | 3 | 7 | 10 |
| Blood cholesterol increased | 0 | 2 | 2 |
| Blood thyroid stimulating hormone decreased | 0 | 1 | 1 |
| International normalised ratio increased | 0 | 1 | 1 |
| *Vital signs* |  |  |  |
| Body temperature increased | 3 | 10 | 13 |
| Body temperature decreased | 0 | 3 | 3 |
| Respiratory rate decreased | 0 | 1 | 1 |
| Respiratory rate increased | 0 | 3 | 3 |
| **Injury, poisoning and procedural complications** |  |  |  |
| Haematoma after vascular puncture | 0 | 2 | 2 |
| Tongue injury | 1 | 0 | 1 |
| Shunt malfunction | 0 | 1 | 1 |
| Postoperative wound complication | 1 | 0 | 1 |
| Post procedural haemorrhage | 1 | 0 | 1 |
| Fall | 2 | 5 | 7 |
| Endotracheal intubation complication | 0 | 1 | 1 |

**Supplementary figure**. Comparison of GOSE scores from patients treated with EDV2209 (cohorts 4 and 5 evaluated at 3 mo. post-SAH) with historical control values published by Trizio et al.10. The proportion of patients with a favorable outcome (GOSE scores of 5-8) are compared to those with an unfavorable outcome (GOSE scores of 2-4)

1. Wilcoxon Signed Rank Test. 2-sided.
2. Wilcoxon Rank Sum Test. 2-sided.
